# Capsaicin and Its Combination with Oleic Acid Affect Membrane Fatty Acid Remodeling and Cytokine–Chemokine Secretion in HepG2 Cells

**DOI:** 10.3390/ijms26178242

**Published:** 2025-08-25

**Authors:** Claudio Tabolacci, Gessica Batani, Stefania Rossi, Daniela Andrei, Maria Bellenghi, Francesca Pedini, Carlo Mischiati, Maria Luisa Scattoni, Mauro Biffoni, Francesco Facchiano, Carla Ferreri, Anna Sansone

**Affiliations:** 1 Department of Oncology and Molecular Medicine, Istituto Superiore di Sanità, Viale Regina Elena 299, 00161 Rome, Italy; claudio.tabolacci@iss.it (C.T.); stefania.rossi@iss.it (S.R.); francesca.pedini@iss.it (F.P.); mauro.biffoni@iss.it (M.B.); francesco.facchiano@iss.it (F.F.); 2 Research Coordination and Support Service, Istituto Superiore di Sanità, Viale Regina Elena 299, 00161 Rome, Italy; marialuisa.scattoni@iss.it; 3 Institute for Organic Synthesis and Photoreactivity (ISOF), National Research Council (CNR), Via P. Gobetti 101, 40129 Bologna, Italy; gessicabatani@cnr.it (G.B.); carla.ferreri@cnr.it (C.F.); 4 Department of Chemistry, Dominican University, 7900 West Division Street, River Forest, IL 60305, USA; dandrei@dom.edu; 5 Center for Gender-Specific Medicine, Gender Prevention and Health Section, Istituto Superiore di Sanità, Viale Regina Elena 299, 00161 Rome, Italy; maria.bellenghi@iss.it; 6 Department of Neuroscience and Rehabilitation, University of Ferrara, Via Luigi Borsari 46, 44121 Ferrara, Italy; msc@unife.it

**Keywords:** membrane fatty acid remodeling, capsaicin, oleic acid, inflammatory mediators, lipidome, PUFA, liposome models, biomimetic experiments

## Abstract

Capsaicin, the main pungent component of chili peppers, exhibits several bioactive properties, such as modulation of adipogenesis and inhibition of liver steatosis by reducing cytoplasmic lipid accumulation. However, no evidence is currently available regarding its effect on the membrane fatty acid remodeling. In this work, we focused on the HepG2 cell model, commonly employed for hepatotoxicity, to examine the lipidome changes after treatment with capsaicin (10 µM), and its combination with oleic acid (100 µM), following the effects after 1.5, 3, and 24 h. In addition, cell viability, lipid accumulation, and secretion of inflammatory mediators were assessed. Notably, the combination of capsaicin with oleic acid completely reverted the correlation between cytokine/chemokine levels and omega-6/omega-3 fatty acids compared to capsaicin alone. Additionally, the combined treatment influenced the protective effect of capsaicin against polyunsaturated fatty acids, as investigated through biomimetic experiments.

## 1. Introduction

Phospholipids, the essential structural components of the plasma membrane bilayers, regulate key biophysical properties such as fluidity and permeability. They are crucial for positioning protein receptors, mediating membrane organization and conformational changes, cell signaling, and intracellular trafficking [1,2]. A clear understanding of the mechanisms involving membrane lipids is essential to unveil molecular processes in both physiological and pathological conditions. Membrane lipidomics provides insights into the dynamics of phospholipid membranes connected to metabolism, diet, stress, nutraceuticals, and drug supplementation [3]. There is a considerable interest in understanding how the phospholipid membranes remodel in response to the interaction between xenobiotics or nutraceuticals [4]. This concept is fundamental for drug discovery, where plasma membrane phospholipids act as direct targets or synergists of synthetic and natural product effects [5]. Our previous studies explored the remodeling of fatty acid phospholipid membranes in response to external stimuli, driven by bioactive molecules through the well-known Lands’ cycle [6,7,8,9,10,11]. In this work, we focused on capsaicin (CAP), chemically identified as N-vanillyl-8-methyl-1-nonenamide, the main pungent component of chili peppers (*Capsicum* spp.) that acts principally as a selective exogenous agonist of the transient receptor potential vanilloid type 1 (TRPV1) through which it exhibits its bioactive properties [12]. CAP exerts analgesic activity [13]; many studies also describe its beneficial effect on metabolic disease by modulating the inflammatory response, insulin sensitivity, and glycemia regulation. Moreover, CAP possesses antioxidant, antimicrobial, anti-obesity, and anticancer properties [14,15,16]. In particular, the anti-obesity property is likely connected with the capacity to stimulate the sympathetic nervous system, reducing energy intake and consumption by fat oxidation through a catecholamine-mediated mechanism [17]. CAP is known to prevent non-alcoholic fatty liver diseases (NAFLDs) by modulating adipogenesis and liver steatosis and inhibiting neutral cytoplasmic lipid accumulation [18,19]. Here, we used the HepG2 liver cancer cell line that is commonly used to study drug metabolism and hepatotoxicity [20,21]. In fact, HepG2 cells maintain many hepatocyte-like functions and therefore represent a suitable model for studying hepatic steatosis, lipotoxicity, and early inflammatory responses in NAFLD [22]. In particular, these cells treated with non-esterified fatty acid, specifically oleic acid (OA) and palmitic acid, can accumulate intracellular triglycerides and form lipid droplets, mirroring hepatic steatosis (the key feature of NAFLD) [23]. The present study was designed to better investigate the well-known lipid-lowering effect of CAP [24] on an experimental model of hepatocellular steatosis after OA exposure in search of new features involving lipids and their pathways. In particular, evidence of CAP’s impact on phospholipid cell membranes is unknown, and data on the role of CAP as a biophysical membrane modulator are obtained only through the liposome models [25]. For this purpose, we obtained the fatty acid membrane profiles of HepG2 cells treated with CAP and evaluated the remodeling also in the presence of OA, being the suitable agent used to induce steatosis in cellular models as previously indicated [26,27,28]. Furthermore, since several diseases, including obesity, are characterized by altered lipid metabolism and dysregulated immune response [29,30,31], lipid accumulation and cytokine/chemokine secretion were assessed and correlated with omega-6 and omega-3 fatty acid content. Additionally, biomimetic experiments were performed by using the liposomal models to estimate the modulation of CAP to protect polyunsaturated fatty acids due to its well-known antioxidant property [32] when it is combined with OA, and the results from the HepG2 cellular model were compared with those obtained from the biomimetic ones. To the best of our knowledge, we are the first to apply membrane lipidomics to study the membrane remodeling induced by CAP in HepG2 cells.

## 2. Results

### 2.1. Effect of Capsaicin on Oleic Acid-Induced Hepatic Steatosis HepG2 Cell Model

**Figure 1:**
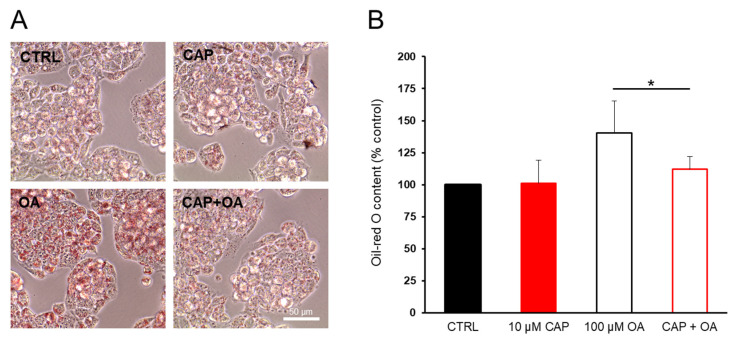
Oil Red-O staining of HepG2 cells. Capsaicin (CAP) counteracts lipid accumulation induced by oleic acid (OA) supplementation. Cells were starved for 24 h and then incubated in DMEM/FFA-free BSA containing OA (100 µM) or CAP (10 µM) or both for 24 h. (**A**) The HepG2 cells were stained with Oil Red-O and observed by a microscope (original magnification: 100×). (**B**) Representative graphs showing intracellular lipid accumulation in HepG2 cells. The relative levels of lipids and fatty acids were quantified by extracting the Oil Red-O dye and by measuring relative absorbance at 490 nm. The bar graph shows the percentage (±SD) of lipids and fatty acids (CTRL = 100%). Statistical significance: * *p* < 0.05.

HepG2 cells have been widely used as a model of NAFLD [33] after exposure to free fatty acids (FFAs) in culture medium [34,35,36]. We first tested the effect of CAP on HepG2 cells. As shown in Figure S1, CAP exerts a general dose-dependent antiproliferative activity on HepG2 cells. Since 10 µM CAP had a slight effect on cell viability, this concentration was chosen for subsequent experiments. A minimal dose was selected to investigate the early and specific effects of CAP on lipid accumulation while avoiding potential off-target effects or cytotoxicity associated with higher concentrations. Oil Red O staining was used as a well-established method to measure the effects of CAP on intracellular neutral lipid accumulation, allowing visualization of lipid droplets within cells. Therefore, intracellular lipid droplets were evaluated under a light microscope. The treatment with CAP decreased the staining of intracellular lipid droplets in OA-treated cells (Figure 1A). Particularly, it can be observed how the intensity of the staining in cells treated with OA alone is reduced by the combined treatment with CAP, reaching levels comparable to those observed in the control (CTRL) or CAP alone. The CAP-dependent suppression of lipid accumulation in HepG2 cells was also confirmed by Oil Red-O quantitation (as described in Section 4) (Figure 1B).

### 2.2. Lipidomic Profile

#### 2.2.1. Membrane Fatty Acid Profile of HepG2 Cellular Model

**Figure 2:**
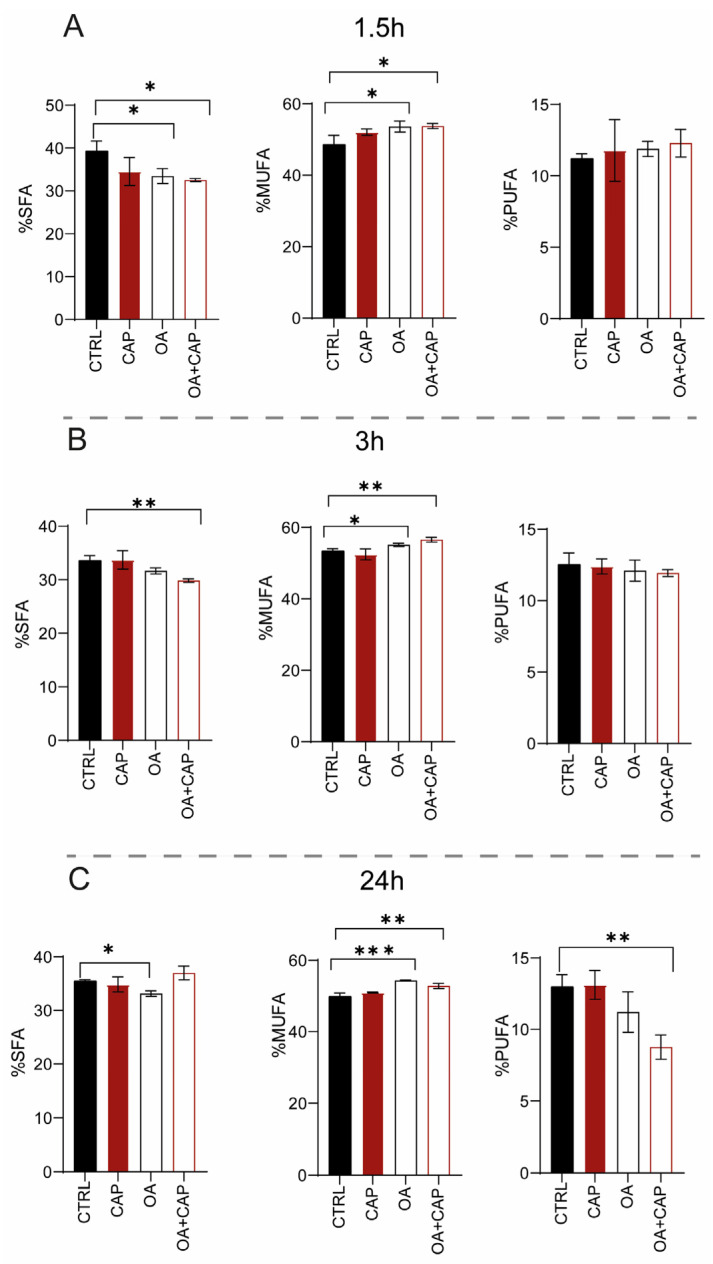
SFA, MUFA, and PUFA families expressed as relative percentage of the quantitative analyses obtained from the phospholipid membranes of HepG2 cell samples treated with 10 µM CAP, 100 µM OA, and mixture (OA and CAP) for (**A**) 1.5 h, (**B**) 3 h, and (**C**) 24 h. The bar graph shows the percentage (±SD). The statistical significance was carried out by comparing each treatment with CTRL; * *p* ≤ 0.05; ** *p* ≤ 0.01; *** *p* ≤ 0.001; F ≥ 5.12 for the significant *p* values.

The membrane fatty acid profiles (the list of fatty acids analyzed, including their nomenclature, is provided in Supplementary Materials) of HepG2 cells were analyzed after treatment with 10 µM CAP or 100 µM OA, or their combination for 1.5 h, 3 h, and 24 h to assess the lipidome modifications at different time points. The fatty acid remodeling was statistically evaluated by comparing the fatty acid profiles of the incubated cells with those without supplementation by using the same conditions and incubation times in triplicate. The membrane isolation, FAME (fatty acid methyl esters) preparation, and GC/GC–MS characterization were performed as previously described [6,37]. Exposure to CAP for 1.5 h did not induce significant changes (Table S1). In the effect of OA, there was a fast incorporation of significantly higher levels of the fatty acid (*p* = 0.0005), which was also reflected in the total monounsaturated fatty acids (MUFAs)’ increase (*p* = 0.012) (Figure 2A).

Moreover, OA supplementation induced a significant decrease in stearic acid (18:0) and in general of saturated fatty acids (SFAs) (*p* = 0.004 and 0.035, respectively) (Figure 2A). It is important to notice that the loss of di-homo-gamma-linolenic-acid (DGLA omega-6) balanced the increase in polyunsaturated fatty acids (PUFAs) omega-3, although this variation was not significant (see Table S1).

**Figure 3:**
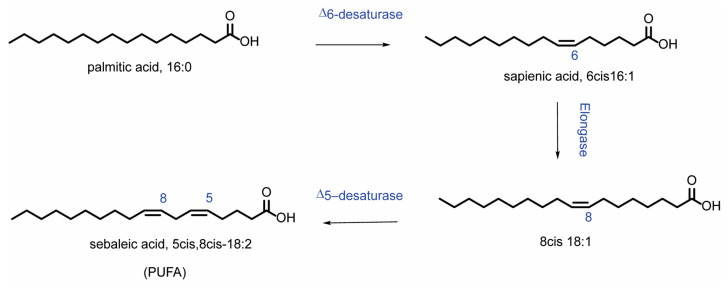
Metabolism of sapienic acid (6*cis* 16:1) starting from palmitic acid. The metabolites of sapienic acid are formed through the following steps: elongation to 8*cis* 18:1 and subsequent desaturation to 5*cis*, 8*cis* 18:2 (sebaleic acid).

With the combined treatment (OA and CAP), in addition to the expected incorporation of OA in the membrane (*p* = 0.0013 compared to CTRL), an increase in myristic acid (14:0) (*p* = 0.0082) and palmitoleic acid (9*cis* 16:1) was also observed. The increase in MUFA content (*p* = 0.017) was balanced by a significant reduction in SFA (*p* = 0.017) (Figure 2A), due to the decrease in palmitic acid (16:0) and stearic acid (*p* = 0.0081 and *p* = 0.023, respectively) (see Table S1). After 3 h of incubation (Table S2), CAP induced not only a significant decrease in sapienic acid (6*cis* 16:1, 16:1 omega-10) (*p* = 0.029) and linoleic acid (18:2 omega-6) (*p* = 0.035), but also an increase in OA, although without significance. The incubation with OA induced its incorporation into membranes (*p* ≤ 0.0001) and increased the content of MUFA (*p* = 0.0150) (Figure 2B). Simultaneously, a decrease in the total content of SFA (Figure 2B), palmitoleic acid (*p* = 0.0005), and *cis* vaccenic acid (*p* = 0.0023) was observed. Treatment with both molecules induced (i) the loss of SFA (*p* = 0.0058) (Figure 2B), due to the decrease in myristic acid and palmitic acid (*p* = 0.0165); (ii) the increase in OA (*p* ≤ 0.0001) and its elongation products, gondoic acid (11*cis* 20:1) (*p* = 0.032), and erucic acid (13cis 22:1) reflected in the total MUFA content (*p* = 0.0012) (Figure 2B). On the contrary, a significant decrease in sapienic acid (*p* = 0.0001), palmitoleic acid (*p* ≤ 0.0001), *cis* vaccenic acid (11*cis* 18:1) (*p* = 0.0006), and linoleic acid (*p* = 0.0005) was observed, whereas the total content of omega-10 was significantly reduced (*p* = 0.023). Interestingly, at 3 h (also noticed in the results at 24 h, Table S3), all treatments modulated the content of sapienic acid and its metabolites: 8*cis* 18:1 (18:1 omega-10) and sebaleic acid (5*cis*, 8*cis* 18:2, 18:2 omega-10) [6,37]. To the best of our knowledge, this is the first report regarding the omega-10 detection in the HepG2 cell model (Figures S2 and S3). Figure 3 describes the omega-10 family pathway.

Table S3 shows the effect of the treatments at 24 h. In detail, the incubation of HepG2 cells with CAP did not induce an evident and significant membrane remodeling. As expected, the treatment with OA confirmed its significant incorporation into membranes (*p* = 0.0001). Moreover, we observed its elongation to gondoic acid (*p* = 0.0027), as well as the increase in the total MUFA content (*p* = 0.0001) (Figure 2C). However, sapienic, palmitoleic, and *cis* vaccenic fatty acids were significantly decreased (*p* = 0.0011, *p* = 0.0001, and *p* = 0.0006, respectively). This effect can be explained by the negative feedback on the desaturase enzyme activity promoted by OA supplementation as previously reported [38,39]. Furthermore, the content of SFA (*p* = 0.0017) (*p* = 0.044) also decreased (Figure 2C). In addition, a significant decrease in docosahexaenoic acid (DHA) omega-3 (*p* = 0.034) and omega-10 levels (*p* = 0.0167), along with an increased omega-6/omega-3 ratio (*p* = 0.027), was noticed.

The combined treatment of OA and CAP showed even more significant effects. On the one hand, the incorporation of OA (*p* ≤ 0.0001) and its metabolite 11*cis* 20:1 (*p* = 0.0078), along with an increase in palmitic acid (*p* = 0.0109), was observed. On the other hand, the myristic and stearic acids were diminished (*p* = 0.0002 and *p* = 0.0011, respectively) together with sapienic acid (*p* = 0.001), palmitoleic acid, *trans* 16:1 (*p* ≤ 0.0001), and *cis* vaccenic acid (*p* ≤ 0.0001). In contrast to the previous incubation times, a significant loss of PUFA was observed (*p* = 0.0032) at 24 h (Figure 2C). Specifically, a significant reduction was observed in omega-6 fatty acids (*p* = 0.0052), particularly in linoleic acid (18:2 omega-6; *p* = 0.025), dihomo-γ-linolenic acid (DGLA; *p* = 0.0051), and arachidonic acid (*p* = 0.0092). This decrease was also detected in omega-3 fatty acids (*p* = 0.0022), specifically in docosapentaenoic acid (DPA; *p* = 0.0086) and docosahexaenoic acid (DHA; *p* = 0.0004). The omega-6/omega-3 ratio was significantly altered compared to the control group (*p* = 0.027). Furthermore, the omega-10 fatty acids were significantly reduced (*p* = 0.0009).

#### 2.2.2. PUFA Evaluation in Biomimetic Models

We believe that a longer exposure to OA (24 h) could strongly influence the biophysical membrane properties, such as the fluidity, as previously reported [40]. Therefore, CAP in the presence of OA could diminish the hydrophobic interaction with fatty acid chains [41,42] by promoting a decreased protection of PUFA moieties as reflected in their loss during the incubation. This effect was not noticed when only CAP was used at the same time of incubation. To prove this hypothesis, we performed the biomimetic experiments by incubating unilamellar liposomes, since they are well-known as representative cellular models [43]. The biomimetic experiments were performed by using the same treatments as for HepG2 cells (10 µM CAP, 100 µM OA, or their combination). We evaluated the modulation of the known antioxidant property of CAP to PUFA [44] in combination with OA. The PUFA protection activity was assessed by using liposomes made of 1-palmitoyl-2-linoleoyl-sn-glycero-3-phosphocholine and by measuring the loss of linoleic acid [43,45,46] based on the ratio value of linoleic acid (18:2) to palmitic acid (16:0). As a liposome model, we employed 1-palmitoyl-2-linoleoyl-sn-glycero-3-phosphocholine, because the fatty acid residue of palmitic acid (saturated fatty acid) is chemically stable to oxidative condition, while the fatty acid residue of linoleic acid (omega-6 PUFA) reacts to oxidation. Therefore, palmitic acid, unaffected by oxidation, can be used as the internal standard to measure the linoleic acid reactivity in the model [43]. As shown in Table 1, we compared the results obtained by using empty liposomes (1 mM in PBS, pH 7.4) incubated with CAP, OA, and their combination in “open air” at 37 °C for 24 h with those obtained by incubating oleic acid-entrapped liposomes (OA-liposomes) with CAP (10 µM) under the same conditions.

In all experiments (performed in triplicate), the empty liposomes (1 mM at the starting time and not incubated) were used as a control, where the reference value of the ratio linoleic acid/palmitic acid was equal to 1, representing 100% of linoleic acid. The results showed that only CAP provided the total protection of linoleic acid without any loss. In contrast, its formulation with OA induced a 19% loss of linoleic acid, showing only partial protection compared with the 62% linoleic acid loss observed with OA alone. When OA-liposomes were incubated (linoleic acid loss equal to 26%), CAP was able to protect 84% of linoleic acid, displaying a result comparable to that observed after the treatment of the CAP and OA mixture in empty liposomes (protection equal to 81%, Table 1). Our results from the experiments conducted using the liposomal suspension model showed that the protective effect of CAP on linoleic acid is critically dependent on its localization and hydrophobic interaction with the fatty acid. These interactions, however, can be disrupted by OA, which increases membrane fluidity and alters the biophysical properties of the liposomal system. To further investigate the critical role of the physical state, we performed a complementary experiment using linoleic acid methyl ester in isopropanol to eliminate the aqueous compartmentalization and the hydrophobic interaction between CAP and the fatty acid chains [41,42]. This experiment enabled us to assess PUFA protection by CAP in solution, where OA cannot interfere with the biophysics of the system, like in the case of the liposomal model. Specifically, a solution of 1 mM linoleic acid methyl ester in isopropanol was incubated with only CAP (10 µM) and its mixture with OA (100 µM) in open-air conditions at 37 °C, for only 6 h to avoid solvent evaporation. Therefore, in this experiment, a homogenous solution of linoleic acid methyl ester was used instead of a liposomal suspension. By using this solution, the presence of OA in the mixture with CAP did not influence the protective effect of CAP to linoleic acid (see Table S4).

Furthermore, we also considered that CAP could lose its protective interaction with PUFA when mixed with OA, due to the spontaneous organization of OA into micelles under our experimental conditions that could sequester CAP [47,48]. Therefore, by using Dynamic Light Scattering (DLS), we monitored the formation of OA or OA and CAP micelles when their solutions were dispersed in PBS buffer at pH 7.4. At the starting time (0 time), the size of OA micelles was equal to 244.88 ± 3.35 nm (polydispersity 0.220), while OA and CAP micelles measured 197.12 ± 6.14 nm (polydispersity 0.226). Our interest was specifically focused on monitoring the micelles at 24 h at 37 °C, as we observed a decrease in PUFA levels both in the cell model and in the biomimetic experiments. At this time, DLS could no longer detect nanoparticle micelles but instead indicated the presence of aggregates (size 11,005.15 ± 3670 nm, polydispersity 10,582). These findings suggest that the protective effect of CAP is influenced by its physical state and membrane environment.

#### 2.2.3. Triglyceride Fatty Acid Profile

**Figure 4:**
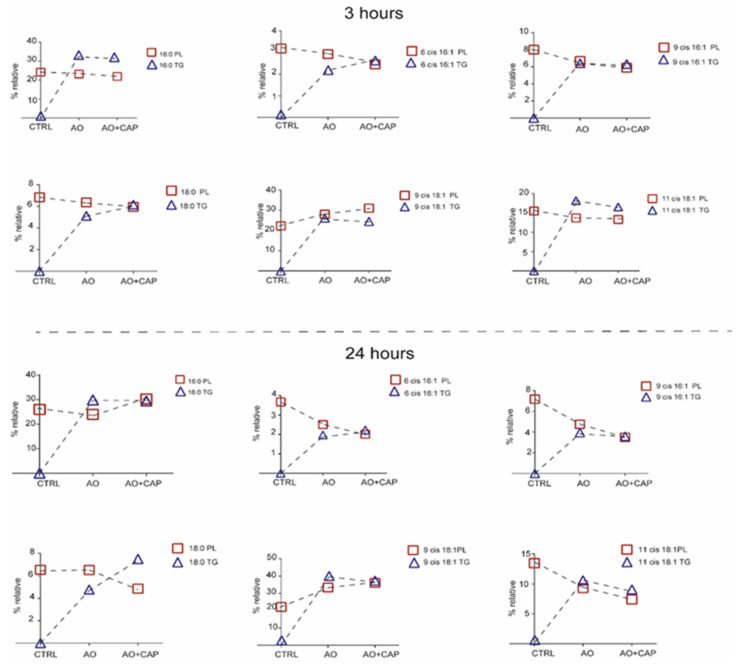
Certain significant fatty acid changes in membrane phospholipid and cytoplasmic triglyceride (TG) composition after treatment with OA/OA with CAP. The significant values of fatty acids of TG are reported in Tables S5 and S6.

In addition to the fatty acid profiles of phospholipid membranes, we also characterized the fatty acid content of the cytoplasmic triglycerides (TGs), since at 3 h and 24 h, lipid droplets were evidenced by TLC after treatments with OA and the combination of OA with CAP (Tables S5 and S6). We compared the fatty acid profiles of TG formed after OA with CAP supplementation with those formed after OA treatment. We observed that both non-treated HepG2 cells and those treated with CAP were free of lipid accumulation. The formation of triglycerides after exposure to OA confirmed the lipid accumulation detected and monitored by Oil Red-O staining (see Figure 1). After 3 h, the fatty acid composition of TG was characterized by an increase in stearic acid (*p* ≤ 0.0001), sapienic acid (*p* = 0.03), omega-10 fatty acid family (*p* = 0.04), and DGLA omega-6 (*p* = 0.009). This effect was balanced by a decrease in total MUFA (*p* = 0.038), particularly OA (*p* = 0.034) and *cis* vaccenic acid (*p* = 0.0071). After 24 h treatment, the total SFA (*p* = 0.018) and the myristic and stearic acids content increased (*p* values 0.0003 and 0.0020, respectively). Conversely, MUFA decreased (*p* = 0.016), specifically OA (*p* = 0.077), *cis* vaccenic acid (*p* = 0.0063), and DHA omega-3 as PUFA (*p* = 0.04). The fatty acid distribution between phospholipid membranes and cytoplasmic accumulations after the treatments can be seen in Figure 4, where certain significant fatty acid changes in membrane phospholipid and cytoplasmic triglyceride composition were highlighted.

Interestingly, after 3 h of incubation, sapienic, palmitoleic, and stearic acids reached similar values between PL and TG, showing an equilibrated content between the two cellular compartments. A different trend occurred for palmitic and cis vaccenic acids, where the fatty acids remained in the form of lipid accumulation in the cytoplasmic environment rather than enriching the phospholipid membranes. OA increased in membrane phospholipids and decreased in TG accumulation. Our results support the evidence that the presence of CAP in the mixture prevents the further accumulation of OA [19,49,50,51], which was consequently incorporated in phospholipid membranes. After 24 h, the cytoplasmic and membrane portioning showed an equal level for palmitic, sapienic, and palmitoleic acids. On the contrary, stearic acid accumulated more in the cytoplasmic lipid droplets rather than in the phospholipid membranes after OA with CAP treatment. The supplementary accumulation of OA in lipid droplets was avoided by merging an equilibrated content with phospholipid membranes, while the *cis* vaccenic acid decreased in PL and TG.

#### 2.2.4. HepG2 Membrane Lipid Class Characterization

The lipid classes of cell membranes were evaluated by using HPLC-MS after 24 h of treatment. We monitored the content of the following components: phosphatidyl glycerol (PG), phosphatidyl inositol (PI), cholesterol (CHO), phosphatidyl serine (PS), phosphatidyl ethanolamine (PE), sphingomyelin (SM), and phosphatidylcholine (PC). As shown in Table 2, CAP induced a significant increase in the content of PS (*p* = 0.0181), while PE and SM were significantly decreased (*p* = 0.0003; *p* = 0.05) with respect to CTRL.

The OA treatment induced a significant decrease in PE content (*p* = 0.012), whereas the combined treatment (OA and CAP) mirrored the effect of CAP alone, increasing the PS content (*p* = 0.0078) and decreasing PE (*p* = 0.0002) content; also, SM showed a decreasing trend, although without significance.

### 2.3. Effects of Capsaicin and Oleic Acid on Inflammatory Mediators

**Figure 5:**
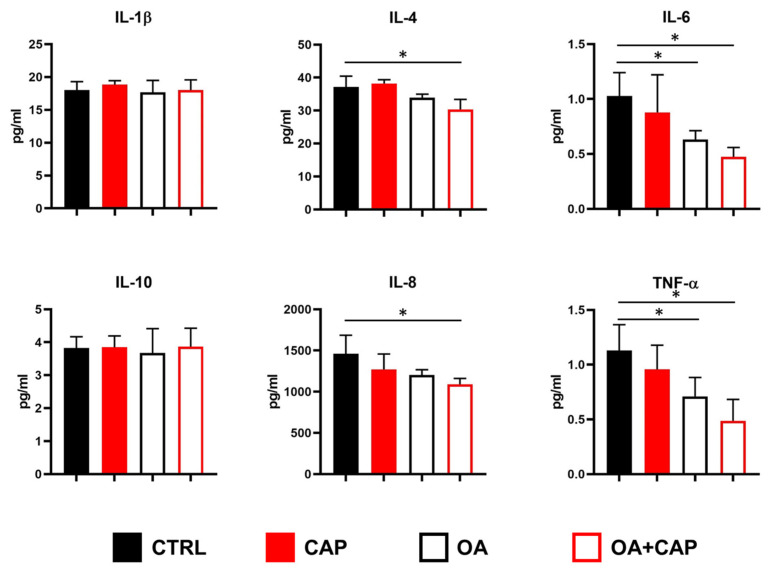
Modulation of cytokines/chemokines secretion in HepG2 cells treated with 10 µM capsaicin (CAP) alone or in combination with 100 µM oleic acid (OA). Concentrations of the indicated analytes in conditioned media were quantified by multiplex immunoassay. The results are shown as mean ± SD (statistical significance versus control cells: * *p* < 0.05; F ≥ 4.6 for the significant *p* values).

It has been demonstrated that CAP regulates the inflammatory response in TRVP1-dependent and TRVP1-independent manner [52,53]. Moreover, adiposity is tightly related to pro-inflammatory cytokines such as interleukin (IL)-1β, IL-6, and tumor necrosis factor α (TNF-α) [54]. Therefore, we decided to measure using Luminex technology, in addition to IL-1β, IL-6, and TNF-α, also the levels of the pro-inflammatory chemokine IL-8 (CXCL8), and two other cytokines, namely, IL-4 and IL-10 (with general anti-inflammatory activity) [55]. As shown in Figure 5, some differences were observed after treatments. In particular, OA significantly reduced the levels of IL-6 and TNF-α, as also observed when combined with CAP. Moreover, drugs combination also reduced the levels of IL-4 and IL-8 (Figure 5).

### 2.4. Multivariable Analysis Identifies Cytokine–Chemokine and PUFA Associations

**Figure 6:**
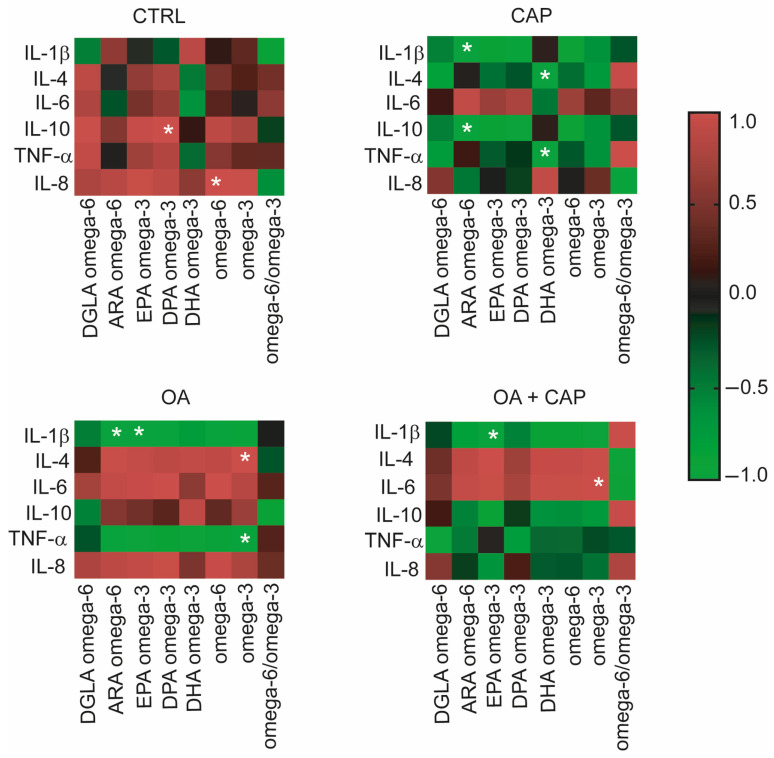
Heatmaps show the correlation matrices between PUFA omega-6 and omega-3 values and cytokine levels in the HepG2 cell model under treatments. The results of the multiple variable analyses based on correlation matrices were expressed as positive or negative coefficients of correlation r and their *p* values; color code reported in the legend spans from red positive r to green negative r; the white asterisks indicate the r coefficients with statistical significance detailed in the corresponding Section 2.

The potential relationship between inflammatory mediators and the fatty acids in our experimental model under the various treatments with CAP, OA, and their combination was investigated. We assessed the multivariable comparison [6] between cytokine levels, omega-3 and omega-6 PUFAs, precursors of anti-inflammatory and pro-inflammatory prostaglandins (PGE3 and PGE2) [56]. Specifically, we performed a statistical multivariable comparison analysis based on correlation matrices by using the cytokine–chemokine levels shown in Figure 6 and the omega-6 and omega-3 values reported in Table S3. The results of this multivariable analysis were graphically represented by the corresponding heatmaps (Figure 6).

Interestingly, in the control cells, IL-10 and IL-8 were positively correlated with DPA (docosapentaenoic acid) omega-3 (*p* = 0.044) and omega-6 (*p* = 0.020) in a significant way. CAP has induced an inverse correlation between cytokines and PUFA compared to the control cells. In fact, we observed a significant negative correlation between ARA (arachidonic acid) omega-6 and IL-1β (*p* = 0.006) and IL-10 (*p* = 0.001), which were positively correlated in the control. Furthermore, we observed a negative correlation between DHA (docosahexaenoic acid) omega-3 and IL-4 (*p* = 0.037) and between DHA omega-3 and TNF-α (*p* = 0.045), the latter becoming significant compared to the control. Additionally, four significant correlations were found in OA-treated cells. Specifically, IL-1β showed negative correlations with ARA omega-6 and EPA (eicosapentaenoic acid) omega-3 (*p* = 0.039 and *p* = 0.037, respectively), while omega-3 was positively correlated with IL-4 (*p* = 0.006) and negatively correlated with TNF-α (*p* = 0.006). By analyzing the correlations in cells treated with CAP, OA, and their combination, it was observed that IL-1β showed a significant negative correlation with EPA omega-3 (*p* = 0.045), while IL-6 was positively correlated with PUFA omega-3 (*p* = 0.036).

## 3. Discussion

The worldwide increase in obesity represents a significant health challenge due to its strong association with insulin resistance, cardiovascular disease (CVD), and increased risk of several cancers. Moreover, obesity (particularly abdominal obesity), along with dyslipidemia, hyperglycemia, and hypertension, is a key component of metabolic syndrome [57]. Chili peppers have been traditionally used for flavoring, food preservation, and medicinal purposes. Several studies highlight that chili peppers, and capsaicinoids as their main active compounds, may help reduce energy intake by promoting a sense of satiety [58]. Therefore, in this work, we studied the effects of CAP in a dyslipidemic cellular model (HepG2 treated with OA). We first demonstrated that CAP showed a dose-dependent antiproliferative effect on HepG2 cells, and we selected the 10 µM concentration for further experiments due to its slight impact on cell viability. Moreover, CAP 10 µM was found to reduce intracellular lipid droplet accumulation in cells treated with OA. To further elucidate the lipid pathways involved, we performed lipidomic profiling of HepG2 cells treated with CAP, OA, or their combination at 1.5, 3, and 24 h. This time-course analysis was designed to follow up the lipidome changes compared to untreated cells, with a specific focus on fatty acid remodeling in both membrane phospholipids and cytoplasmic triglycerides (see Figure 2 and Figure 4). Fatty acid content was quantified using a previously validated and highly accurate analytical protocol [6]. First of all, we observed that by performing the lipidomic analysis, we found the omega-10 fatty acid family in the starting HepG2 cells, reporting the presence for the first time in this type of cell. The metabolic pathways of these fatty acids are illustrated in Figure 3. Interestingly, following treatment with CAP and OA + CAP at 3 h, a significant decrease in 16:1 omega-10 (6*cis*-16:1, sapienic acid) and in total omega-10 fatty acid content was observed in membrane phospholipids, accompanied by a parallel increase in the cytoplasmic triglyceride fraction (see Figure 4, Tables S2 and S5). A similar trend was also observed at 24 h following the combined treatment (see Tables S3 and S6), affecting both membrane phospholipids and triglycerides. Additionally, the content of 18:2 omega-10 (5*cis*, 8*cis*-18:2, sebaleic acid) was also significantly decreased at 24 h in response to the combined treatment.

Notably, sebaleic acid (5*cis*, 8*cis* 18:2) represents the first detected PUFA endogenously produced by mammal cells; from chemical perspective, it is a positional isomer of linoleic acid (9*cis*, 12 *cis* 18:2). It is well known that mammal cells have to get the two essential PUFAs, linoleic acid omega-6 and alpha-linolenic acid omega-3 from external sources, as these are exclusively produced by plants, which have the appropriate desaturase enzymes for their biosynthesis [59]. Only after uptake, these essential fatty acids are endogenously converted by cellular metabolic pathways into long-chain omega-6 and omega-3 fatty acids. In this work, we determined the omega-10 fatty acid content by chemical–analytical strategy (see Section 4), based on the characterization of diagnostic fragments by GC–MS (Figure S3) according to the described procedures [37,60]. As previously published by our group, the omega-10 fatty acid family plays an emerging and interesting role in tumor development [61]. They are present in several tumoral cells, such as Caco-2 colorectal cancer cells [37], prostate PC-3 and LNCaP cancer cells [62], human bronchial epithelial BEAS-2B cells, and relative exosomes [63], as well as in human red blood cells and plasma in obesity-associated disorders [64,65]. We demonstrated that the omega-10 fatty acids were able to modulate the expression and activation of EGFR receptor (epidermal growth factor receptor), AKT (protein kinase B), and mTOR (mammalian target of rapamycin) signaling pathway in MCF-7, MDA-MB-231, and BT-20 breast cancer cell lines [6]. The role of omega-10 fatty acids in cancer is still debated, as well as their possible xenobiotic activity. In a recent study, we demonstrated a pro-cancer effect associated with omega-10 fatty acids [6]. However, research in this area is still ongoing to further elucidate the role of omega-10 fatty acids across different tumor cell types.

**Figure 7:**
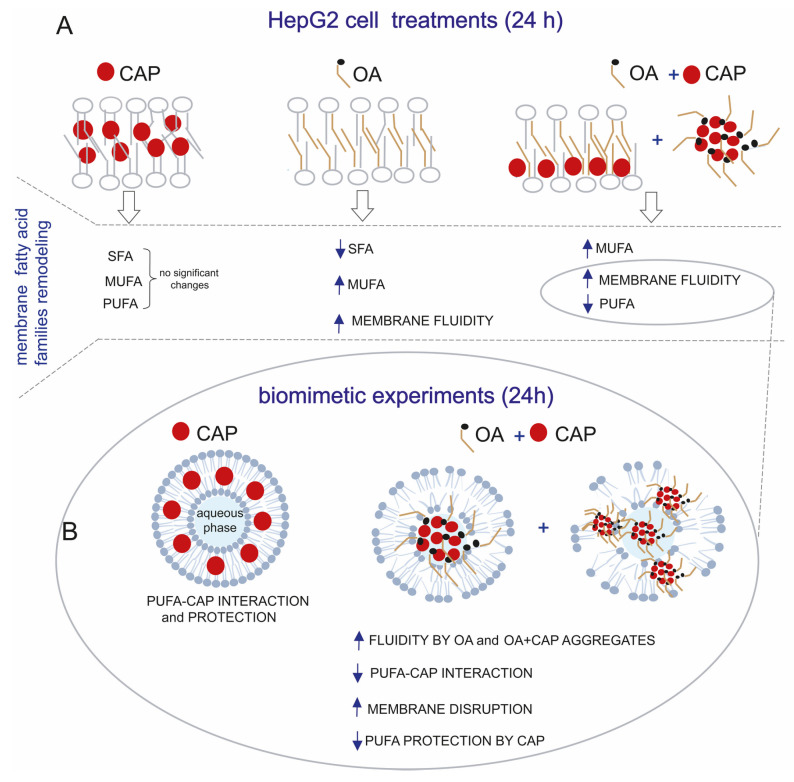
(**A**) Membrane fatty acid families remodeling of HepG2 cells treated with CAP (10 µM), OA (100 µM), and OA + CAP for 24 h. (**B**) The lipidomic profiles were compared with biomimetic experiments performed by incubating liposomes made of PLPC for 24 h in open-air conditions at 37 °C with CAP and OA + CAP. This shows the decrease in PUFA protection by CAP due to loss of CAP–PUFA interaction in more fluid membranes or membranes disrupted by OA and CAP aggregates.

In general, membrane fatty acid remodeling observed at the early time points (1.5 h and 3 h) revealed a significant increase in MUFAs, attributable to the incorporation of oleic acid following OA and OA + CAP treatments. This increase in MUFA content was accompanied by a decrease in SFA, while PUFA remained unchanged (see Figure 2). Interestingly, membrane lipidomics at 24 h revealed that the OA + CAP treatment induced a significant loss of PUFA, which was not observed with CAP alone (see Figure 2 and Table S3). We believe that prolonged exposure to OA (24 h) altered the biophysical properties of the cell membrane, increasing membrane fluidity [40], weakening hydrophobic interactions between CAP and fatty acids [41,42], and consequently reducing the protective effect of CAP on PUFA [44]. To investigate this, we carried out biomimetic experiments by incubating unilamellar liposomes, commonly used as representative cell membrane models, with the same treatments applied to HepG2 cells. In particular, we demonstrated that in the liposomal model composed of 1-palmitoyl-2-linoleoyl-sn-glycero-3-phosphocholine, the PUFA residue of linoleic acid was fully preserved only during the incubation with CAP under “open-air” conditions at 37 °C. In contrast, we observed a decrease in linoleic acid content, with a 19% loss, when CAP was combined with OA (see Table 1). Our results obtained from the liposomal suspension model showed that the protective effect of CAP on linoleic acid is critically dependent on its localization and hydrophobic interaction with the fatty acid due to the chemical structure of CAP [43]. The addition of OA increases membrane fluidity, altering the biophysical properties of the model and generating a “disturbing effect” on the hydrophobic interactions, leading to a loss of PUFA protection by CAP. This phenomenon was observed in a heterogeneous physical system, such as aqueous suspensions, as indicated by our results. To further support this hypothesis, we also performed complementary incubations in a different environment by using a model fully soluble in the experimental system, specifically linoleic acid methyl ester in a solution of isopropanol. Being in solution rather than in an aqueous suspension, this system lacks the hydrophobic interactions between CAP and fatty acid chains. As a result, the incubations with the same treatments, particularly the addition of OA (or CAP + OA), did not introduce a “disturbing element”, did not change the physical state, and did not impair the general PUFA protection by CAP (see Table S4). This highlights that the efficacy of antioxidants against oxidative stress conditions is influenced not only by their intrinsic chemical properties but also by their localization and by the system’s physical conditions. Moreover, we also considered that, under our experimental conditions, the protective interaction between CAP and PUFA could be impaired due to the incorporation of CAP in spontaneously formed micelles with OA. This hypothesis was confirmed by DLS, which indicated the formation of micelles with an average diameter of approximately 200 nm in PBS buffer at pH 7.4. Notably, these micelles evolved into large aggregates (~11,000 nm) after 24 h, a timing point at which the reduction in PUFA levels was observed both in the cellular model and in the biomimetic liposomal system. Considering these experimental results, we concluded that the CAP sequestration in big aggregates might have affected the interaction with PUFA and, therefore, the ability to protect them in membranes. These findings clearly demonstrate how the CAP ability against PUFA oxidation is influenced by its state, aligning with the observations from the cell model, where the CAP and OA mixture promoted a significant loss of PUFA compared to the treatment with only CAP at 24 h. Our biomimetic experiments using liposome suspension mirroring the cell models suggest that CAP may lose its protective interaction with fatty acid chains when combined with certain molecules, such as oleic acid, enhancing membrane fluidity [40,41] or, even more critically, disrupting membrane integrity through aggregate formation, as previously reported [66]. Figure 7 describes the modulation of PUFA protection by CAP in more fluid or altered membranes due to OA by comparing the membrane fatty acid families remodeling of the HepG2 cells (Figure 7A) with the results obtained from the biomimetic experiments (Figure 7B). This outcome is very important for developing formulations and supplements containing antioxidant molecules, for which the beneficial effect could be altered by the other components, excipients, and physical state.

Another interesting aspect is the profile of the lipid classes present in cell membranes (see Table 2). On the one hand, CAP significantly increased PS levels, while simultaneously reducing PE and SM. On the other hand, OA alone reduced PE, and combined treatment (OA + CAP) similarly to CAP increased PS levels and reduced PE, with a trend toward a non-significant reduction in SM. These findings provided insights into the differential effects of CAP and OA on the lipid composition of cell membranes. It is important to highlight the increase in PS content induced by CAP treatment, which modulates PS levels within cell membranes. PS plays a crucial role in various cellular processes, including cell death and signaling [67,68]. For instance, the pro-apoptotic effect of CAP on the HepG2 cell line has been well established at higher concentrations [69]. In our experiments, however, the CAP concentration of 10 µM induced only a slight decrease in cell proliferation (see Figure S1), an effect that may be linked to the observed increase in PS content. These findings are consistent with a previous study showing that CAP significantly increased PS externalization in bone marrow mesenchymal stem cells [70]. Furthermore, the decreased content of SM induced by CAP can lead to an increase in membrane fluidity at a low concentration, aligning with the data previously published, in which CAP at relatively low concentrations is intercalated in phospholipid moieties, perturbing the membrane lipids’ packing and increasing membrane fluidity [71]. The significant decrease in PE is the only common effect observed in all treatments. This observation could suggest that they modulate the PE transformation to N-acyl-phosphatidyl-ethanolamine via N-acyl transferase and N-acyl phosphatidylethanolamine phospholipase D (NAPE-PLD), also in a dose-dependent manner, as well as with time and temperature, in agreement with previously published studies [72,73,74].

It has been demonstrated that dietary CAP reduces obesity-induced inflammation and metabolic dysfunction in obese mice by lowering glucose intolerance, inflammatory cytokines, and macrophage infiltration in adipose tissue and liver [75]. Therefore, we measured a panel of inflammatory mediators in order to investigate the effects of CAP with or without OA in our experimental model. Results showed that OA significantly lowered IL-6 and TNF-α levels, an effect also seen with the OA and CAP combination, which additionally reduced IL-4 and IL-8 levels (see Figure 5). These data suggest that CAP alone is not able to modify the secretion of selected cytokines/chemokines, probably due to the low concentration of the drug in our experimental model. In fact, on the one hand, several studies on LPS-stimulated macrophages provided evidence for the anti-inflammatory properties of CAP, which include the inhibition of IL-1β, IL-6, and TNF-α production [76,77]; on the other hand, it has been also reported that CAP supports IL-6 secretion in human bronchiolar epithelial (BEAS-2B) [78] and in TNF-α-stimulated PC-3 prostate cancer cell line [79]. The observed reduction in pro-inflammatory mediators after combined treatments suggests that CAP might enhance the well-known anti-inflammatory effects of OA [80,81].

Moreover, multivariable comparison analysis between cytokine levels, omega-3 and omega-6 PUFAs, and precursors of anti-inflammatory and pro-inflammatory prostaglandins (PGE3 and PGE2) was conducted to explore their potential relationships (see Figure 6). The statistical correlation between cytokine levels and omega-3 and omega-6 yielded intriguing findings. In general, the metabolite correlation analysis showed that IL-1β had the highest number of correlations, consistently displaying an inverse correlation with PUFA across all treatments compared to control, supporting its potential role in the regulation of lipid metabolism as previously reported [82,83]. For the first time, our data revealed connections between cytokine levels and membrane fatty acids in HepG2 cells treated with CAP and its combination with OA. Some distinct patterns have emerged, indicating differential modulation of cytokine–PUFA relationships across treatment groups. Specifically, we considered the effect of CAP related to IL-4 and IL-10, both well-established anti-inflammatory cytokines [55,84]. The observed significant inverse correlations between IL-4 and DHA omega-3 suggest that an increase in IL-4 level is associated with the formation of PGE3 and DHA-derived resolvins. In contrast, no correlation was found with ARA omega-6. Furthermore, the anti-inflammatory cytokine IL-10 exhibited a significant inverse correlation with ARA omega-6, while no correlation was observed with DHA omega-3. The inverse correlation between increased IL-10 levels and ARA omega-6 may be attributed to the conversion of ARA into pro-inflammatory PGE2 mediators. These mediators were counterbalanced by the production of the anti-inflammatory PGE3 from EPA and DPA omega-3 [85], which were conversely correlated with IL-10, although with no significance. The anti-inflammatory effect was also supported by the significant negative correlations observed between IL-1β and ARA omega-6 and between TNF-α and DHA omega-3. We can conclude that the modulation of the inflammatory effect of the cytokines was correlated with the inhibition of ARA transformation into the inflammatory mediator PGE2, promoted by DHA omega-3. All these outcomes with the CAP treatment suggest that omega-3 PUFA may contribute to the formation of anti-inflammatory lipid mediators, therefore modulating the production of pro-inflammatory cytokines. The inhibition of PGE2 production, a potent pro-inflammatory mediator, by DHA omega-3 has been reported in various cell types, including LPS-stimulated RAW 264.7 cells [86]. Our results are in agreement with the previous evidence, demonstrating that inflammatory cytokines, such as IL-1β and TNF-α, can also be influenced by lipid mediators derived from PUFA, notably PGE2 from ARA omega-6 and PGE3 from EPA and DHA omega-3 [85,87]. Interestingly, the combined treatment of CAP with OA completely reverted the correlation between PUFA and IL-4, compared to CAP alone. Indeed, IL-4 gave a positive correlation with all omega-6 and omega-3 PUFAs, although without significance (see Figure 6). This positive correlation was also evidenced in the treatment with OA alone. Specifically, we noted a significant correlation between IL-4 and the anti-inflammatory precursor omega-3 PUFA. Additionally, omega-3 demonstrated a significant positive correlation with TNF-α, implying a potential anti-inflammatory role of these fatty acids in the presence of oleic acid. The data suggest that OA may exert modulatory effects on cytokine responses through the PUFA pathway. Overall, considering the decreasing trend of the levels of the inflammatory cytokines in treatments (see Figure 5), which were especially significant in the case of molecule combination, could suggest that cytokines were directly modulated by omega-3 PUFA. In fact, the inhibition of pro-inflammatory mediators such as IL-6, TNF-α, PGE2, and nitric oxide by omega-3 fatty acids has been well-documented in the literature [88,89,90].

## 4. Materials and Methods

### 4.1. Materials

Commercially available *cis* and *trans* fatty acid methyl esters (FAMEs), dimethyl disulfide, cholesterol, and a mixture of phospholipids and iodine were purchased from Merck (Darmstadt, Germany) and used without additional purification; the *trans* FAME isomers not commercially available were synthesized as described [91], 6*cis* hexadecenoic acid methyl ester, 7*cis* hexadecenoic acid methyl ester, 8*cis* 18:1 methyl ester, and 5*cis*, 8*cis* 18:2 methyl ester and 1-palmitoyl-2-linoleoyl-sn-glycero-3-phosphocholine were purchased from Lipidox (Lidingö, Sweden). Acetonitrile, chloroform, methanol, diethyl ether, *n*-hexane (HPLC grade), and absolute ethanol were purchased from J.T. Baker (Phillipsburg, NJ, USA) and used without further purification. Analytical thin-layer chromatography (TLC) was performed on Merck silica gel 60 plates, 0.25 mm thickness, and spots were detected by spraying the plate with cerium ammonium sulfate/ammonium molybdate reagent and revealed by heating the plate. Dulbecco’s modified Eagle’s medium (DMEM), glutamine, penicillin (10,000 U/mL) and streptomycin (10.000 mg/mL), phosphate buffer saline (PBS), capsaicin (CAP), oleic acid (OA), fatty acids-free (FFAs) bovine serum albumin (FFA-free BSA), dimethyl sulfoxide (DMSO), Oil Red-O, and all reagents were from Merck (Darmstadt, Germany) unless stated otherwise. Fetal calf serum (FCS) was purchased from Corning Incorporated (Corning, New York, NY, USA). OA was diluted in isopropanol (95% HPLC grade) to obtain a 40 mM stock solution, while CAP was dissolved in ethanol to obtain a 100 mM stock solution.

### 4.2. Cell Culture and Treatments

The human HepG2 cells (from American Type Culture Collection, Manassas, VA, USA; kindly provided by Dr. Claudia Giampietri, Dept. of Anatomy, Histology, Forensic Medicine and Orthopedics, Sapienza University of Rome, Italy), a well-established model for studies on lipid metabolism [20,26], were cultured in DMEM supplemented with 10% FCS, 0.05% L-glutamine, 1% penicillin and streptomycin, and kept at 37 °C in a humidified atmosphere in the presence of 5% CO_2_. For proliferation studies, HepG2 cells were seeded (8 × 10^4^) in 6-well plates in complete medium and treated with different concentrations (10, 50, and 100 µM) of CAP for 24, 48, and 72 h. Cells were then harvested and counted with a Neubauer-modified chamber. To induce lipid accumulation, HepG2 cells were exposed to 100 µM OA. The use of OA is supported by its ability to be rapidly esterified into triglycerides and stored in lipid droplets, effectively mimicking intracellular fat accumulation. In contrast, other fatty acids, such as linoleic acid, are more prone to oxidative stress and may induce cytotoxicity [92]. In detail, the cells were seeded in 100 mm petri dishes in DMEM complete medium. After 24 h, the culture medium was removed, and the cells were washed three times in PBS (without Ca^++^/Mg^++^) to eliminate residual FCS. Then cells were starved for 24 h in serum-free medium. Finally, cells were incubated in DMEM/FFA-free BSA (1%) containing OA (100 µM) or CAP (10 µM) or both for the indicated time.

### 4.3. Oil Red-O Staining

Intracellular lipid content was examined by Oil Red-O staining as previously described [25], with slight modifications. Briefly, a stock Oil Red-O solution was prepared by dissolving 0.7 g Oil Red-O in 200 mL isopropanol, while a working solution was obtained by mixing 6 parts Oil Red-O stock with 4 parts of bi-distilled water [27]. The HepG2 cells were cultured as described in the previous paragraph and treated with OA and/or CAP for 24 h. The cells were then washed three times in PBS and fixed with 4% paraformaldehyde for 30 min. After the incubation, the cells were washed three times with PBS and stained with Oil Red-O solution for 15 min at room temperature. The cells were subsequently rinsed with distilled water, and the samples were lysed in DMSO; the absorbance of extracted dye was read at 490 nm on a MultiLabel Plate Reader VICTOR X3 (PerkinElmer, Norwalk, CT, USA). Additionally, the cells were also analyzed by EVOS xl microscope (AMG, Seattle, WA, USA).

### 4.4. Lipidomic Profiles

Fatty acid methyl esters (FAMEs) were analyzed by GC (Agilent 6850, Cernusco sul Naviglio, Milan, Italy), using the split mode (50:1), equipped with a 60 m × 0.25 mm × 0.25 µm (50%-cyanopropyl)-methylpolysiloxane column (DB23, Agilent, Santa Clara, CA, USA), and a flame ionization detector with the following oven program: The temperature started from 165 °C, held for 3 min, followed by an increase of 1 °C/min up to 195 °C, held for 40 min, followed by a second increase of 10 °C/min up to 240 °C, and held for 10 min. A constant pressure mode (29 psi) was chosen using helium as carrier gas. FAMEs were identified by comparing the retention times of standard references. They are expressed in quantitative relative percentages (mean ± SD) quantified based on calibration curves of standard references as previously reported (36). Dimethyl disulfide adducts of FAMEs were analyzed by GC–MS (Thermo Scientific Trace 1300; Thermo Fisher, Waltham, MA, USA) equipped with a 15 mm × 0.25 mm × 0.25 µm TG-SQC 5% phenyl methyl polysiloxane column with helium as carrier gas coupled to a mass selective detector (Thermo Scientific ISQ; Thermo Fisher. Waltham, MA, USA) with the following oven program: temperature started at 80 °C, maintained for 2 min increased at a rate of 15 °C/min up to 140 °C, increased at a rate of 5 °C/min up to 280 °C, and held for 10 min. The membrane lipid class characterization was performed with an Agilent 1260 Infinity II HPLC system equipped with a 5 µm C18 column (25  ×  4.6 mm) using as eluent: acetonitrile/isopropanol/tri-distilled water + 10 mM ammonium acetate: 30:60:10, detector UV at 203 nm, injection 20 µL, coupled with an InfinityLab single quadrupole Liquid Chromatography/Mass Selective Detector (LC/MSD). Phospholipids and cholesterol were identified by comparing the retention times of commercially available references. The values were expressed in relative quantitative percentages (% rel. quant.), i.e., they were obtained in µg/mL by using the calibration curves and converted to percentages of each lipid class over the total amount of lipid classes taken as 100% (6). The liposome reactions were performed in an incubating orbital shaker (Argolab, Ski 4, Carpi, Italy), at 37 °C. The hydrodynamic diameters of liposomes and micelles were measured using the Dynamic Light Scattering (DLS) technique (Malvern Instruments Series NanoZS, Malvern Instruments, Malvern, UK) with a detection angle of 173°. All measurements were recorded at 25 °C.

#### Lipid Characterization and Fatty Acid Analysis from HepG2 Cells

To the HepG2 cell samples (3 × 10^6^ cells), washed with PBS, 1 mL of tri-distilled water was added, followed by a cycle of centrifugation (14,000 rpm for 15 min at 4 °C). The resulting membrane pellets (resuspended in 1 mL of tri-distilled water) and the aqueous supernatant phases were separately extracted with a 2:1 chloroform/methanol (CHCl_3_/MeOH) solution (4 × 4 mL) using the Folch method [93]. The organic layers of each extraction were collected, dried with anhydrous sodium sulfate (Na_2_SO_4_), and then evaporated to dryness. The lipid extracts obtained from the membrane pellets and the supernatant aqueous phases were analyzed by TLC (eluent *n*-hexane–diethyl ether 9:1) to determine the lipid class composition. The lipid extract (1.03 ± 0.32 mg) of the membrane pellets, which contained phospholipids (PLs) and free cholesterol (CHO), was also characterized using HPLC-MS by dissolving 0.2 mg of extract in 100 µL MeOH [6]. The TLC monitoring of the extract obtained from the supernatant aqueous phases revealed the presence of triacylglycerols (TGs), only at 3 and 24 h (0.6 ± 0.04 mg) [37]. In the next step, the lipid extracts were converted to FAME by adding 0.5 M KOH/MeOH (0.5 mL), followed by 10 min stirring for the extracts containing PL, while those containing TG were stirred for 30 min. Both reactions were performed at room temperature. The selective transesterification reactions were quenched with brine (0.5 mL), and the FAME mixtures were extracted with *n*-hexane (4 × 2 mL), dried with anhydrous Na_2_SO_4_, evaporated to dryness, and then dissolved in *n*-hexane for GC analyses. An example of a GC chromatogram is shown in Figure S2.

### 4.5. Dimethyl Disulfide (DMDS) Derivatization

The derivatization of the FAME mixture from HepG2 cells was performed by using a known procedure [37], and GC/MS was used to assign the double bond position [38]. Specifically, in a Wheaton vial containing FAME dissolved in *n*-hexane (50 μL), dimethyl disulfide (50 µL) and 2 drops of a 6% solution of iodine in diethyl ether were sequentially added. The reaction was stirred at 38 °C for 2.5 h under an argon atmosphere. Then 1 mL of *n*-hexane and 1 mL of a 5% aqueous sodium thiosulfate solution were added. The organic phase was dried with anhydrous Na_2_SO_4_, concentrated under a gentle stream of nitrogen, and injected for GC–MS analysis. An example of DMDS derivatization is reported in Figure S3.

### 4.6. Preparation and Incubation of Biomimetic Models

The liposomes were developed by applying published procedures [43,45,46]. Specifically, in two separate 10 mL round-bottom flasks, 10 mg (0.013 mmol) of 1-palmitoyl-2-linoleoyl-sn-glycero-3-phosphocholine (PLPC) were added. OA (0.37 mg, 0.0013 mmol) was inserted in one of the flasks. The reagents were dissolved in chloroform and evaporated to dryness until a thin lipid film was obtained. The flasks were kept under vacuum for 5 h to eliminate traces of solvent. Then 1.3 mL of tri-distilled water was added to obtain a stock liposomal suspension of 10 mM, followed by stirring at 2500 rpm for 5 min to form multilamellar vesicles (MLVs). The unilamellar vesicles were then prepared by the extrusion technique (LUVET) using a filter of 200 nm diameter. The empty liposomes (final experimental concentration of 1 mM in PBS pH 7.4) were treated according to the cellular model with OA (100 µM), CAP (10 µM), or the mixture of both molecules. The liposomes containing OA (OA-liposomes) (final concentrations 1 mM and 100 µM in PBS pH 7.4) were treated only with CAP (10 µM). The incubations of all suspensions were performed in open-air conditions at 37 °C for 24 h in an orbital shaker, followed by work-up (lipid extraction, transesterification, and GC analysis) as described.

### 4.7. Cytokine/Chemokines Evaluation in HepG2 Conditioned Medium

The conditioned media (CM) of HepG2 cells, treated as previously described, were collected and centrifuged to remove any cell debris. IL-1β, IL-4, IL-6, IL-8 (CXCL-8), IL-10, and TNF-α were quantified in the HepG2 CM using a human magnetic Luminex assay (R&D Systems, Minneapolis, MN, USA) according to the manufacturer’s instructions. The quantification was carried out with a Bio-Plex array reader (Bio-Plex 200 System) equipped with a magnetic washer workstation and Bio-Plex Manager software (Version 6.1 Bio-Rad Laboratories, Hercules, CA, USA).

### 4.8. Statistical Analysis

All the experiments were performed in triplicate (*n* = 3). All the values are expressed as means ± SD. Statistics were calculated with GraphPad Prism 8.0 software (GraphPad Software, Inc., San Diego, CA, USA). The comparisons were conducted by ANOVA analysis followed by Dunnett’s test; two-group comparison was conducted by using a two-tailed Student’s *t*-test. Multiple Variable Analyses were performed between PUFAs, omega-6, and omega-3, and cytokine–chemokine levels. The significance of the comparisons and Multiple Variable Analyses was given as *p*-value ≤ 0.05.

## 5. Conclusions

It has been reported that in a large Mediterranean cohort of adults, regular consumption of chili peppers, where CAP is the main representative bioactive compound, is associated not only with a lower risk of CVD mortality but also with an increased level of circulating lipids (i.e., cholesterol and triglycerides) [94]. Therefore, improving the knowledge of CAP effects on lipid metabolism sparks particular interest. To the best of our knowledge, this is the first literature report where the membrane fatty acid remodeling promoted by CAP was investigated by using the HepG2 cell line, a common model for steatosis. Furthermore, we have observed these effects by treating cells with relatively low doses of CAP, avoiding the apoptotic effects previously observed following exposure to higher doses [71]. Our data demonstrated changes in cytokine levels and their correlation with PUFA omega-6 and omega-3, as well as modulation of the well-known antioxidant property of CAP as a PUFA preservative after treatment with or without OA.

## Figures and Tables

**Table 1:** PUFA protection activity of capsaicin (CAP).

Incubation of Liposomes at 37 °C, 24 h	PUFA Loss% ^1^ (Mean ± SD)
+10 µM CAP	0
+100 µM OA	62.05 ± 0.05
Mixture 10 µM CAP + 100 µM OA	19 ± 0.09
(OA-liposome)	26 ± 0.5
(OA-liposome) + 10 µM CAP	16.14 ± 1.06

^1^ PUFA loss % is calculated on the value of the linoleic/palmitic acid ratio of CTRL (controls) represented by empty liposomes at the starting time, without incubation. OA, oleic acid.

**Table 2:** Main lipid classes isolated from HepG2 cell membranes treated with 10 µM capsaicin (CAP), 100 µM oleic acid (OA), and OA + CAP after 24 h.

	CTRL	CAP	OA	CAP + OA
PG	8.17 ± 0.61	8.85 ± 0.15	7.90 ± 0.40	7.90 ± 0.80
PI	10.17 ± 0.90	10.35 ± 0.45	9.46 ± 1.05	10.80 ± 0.50
CHO	5.96 ± 0.45	5.28 ± 0.53	5.27 ± 0.58	6.00 ± 0.20
PS	13.05 ± 1.25	16.95 ± 0.75 *	15.51 ± 1.15	16.68 ± 1.03 **
PE	18.62 ± 0.23	16.13 ± 0.39 ***	16.58 ± 0.49 **	15.83 ± 0.57 ***
SM	7.35 ± 0.25	6.20 ± 0.30 *	8.28 ± 0.85	6.30 ± 0.40
PC	36.66 ± 1.94	35.93 ± 0.42	36.70 ± 0.50	35.75 ± 0.85

Values are expressed as relative quantitative percentages (% rel. quant.) calculated from the quantities of lipids obtained by HPLC-MS analysis, identified and calibrated by the standard references as described in Materials and Methods. Values are mean ± SD of the experiments performed in triplicate. Statistics were carried out in comparison with control (CTRL) grown under the same conditions without treatments; significance *p* values * ≤ 0.05; ** ≤ 0.01; *** ≤ 0.001; F ≥ 6.8 for the significant *p* values; PG, phosphatidyl glycerol; PI, phosphatidyl inisitol; CHO, cholesterol; PS, phosphatidyl serine; PE, phosphatidyl ethanolamine; SM, sphingomyelin; PC, phosphatidyl choline.

## Data Availability

The data generated in the present study are included in the figures and/or tables of this article and in the Supplementary Materials.

## Supplementary Materials

The following supporting information can be downloaded at https://www.mdpi.com/article/10.3390/ijms26178242/s1.

## References

[1] Renard K., Byrne B. (2021). Insights into the Role of Membrane Lipids in the Structure, Function and Regulation of Integral Membrane Proteins. Int. J. Mol. Sci..

[2] Cournia T.W., Allen I., Andricioaei B., Antonny D., Baum G., Brannigan N.-V., Buchete J.T., Deckman L., Delemotte C., Del Val R. (2015). Membrane Protein Structure, Function, and Dynamics: A Perspective from Experiments and Theory. J. Membr. Biol..

[3] Kostidis S., Sánchez-López E., Giera M. (2023). Lipidomics analysis in drug discovery and development. Curr. Opin. Chem. Biol..

[4] Francia V., Montizaan D., Salvati A. (2020). Interactions at the cell membrane and pathways of internalization of nano-sized materials for nanomedicine. Beilstein J. Nanotechnol..

[5] Escribá P.V., González-Ros J.M., Goñi F.M., Kinnunen P.K.J., Vigh L., Sánchez-Magraner L., Fernández A.M., Busquets X., Barceló-Coblijn G., Torres M. (2015). Membrane Lipid Therapy: Modulation of the Cell Membrane Composition and Structure as a Molecular Base for Drug Discovery and New Disease Treatment. Prog. Lipid Res..

[6] Kuecueksayan E., Sansone A., Chatgilialoglu C., Ozben T., Tekeli D., Talibova G., Ferreri C. (2022). Sapienic Acid Metabolism Influences Membrane Plasticity and Protein Signaling in Breast Cancer Cell Lines. Cells.

[7] Valerii M.C., Turroni S., Ferreri C., Zaro M., Sansone A., Dalpiaz A., Botti G., Ferraro L., Spigarelli R., Bellocchio I. (2021). Effect of a Fiber D-Limonene-Enriched Food Supplement on Intestinal Microbiota and Metabolic Parameters of Mice on a HighFat Diet. Pharmaceutics.

[8] Hanikoglu A., Kucuksayan E., Hanikoglu F., Ozben T., Menounou G., Sansone A., Chatgilialoglu C., Di Bella G., Ferreri C. (2019). Effects of Somatostatin and Vitamin C on the Fatty Acid Profile of Breast Cancer Cell Membranes. Anticancer Agents Med. Chem..

[9] Hanikoglu A., Kucuksayan E., Hanikoglu F., Ozben T., Menounou G., Sansone A., Chatgilialoglu C., Di Bella G., Ferreri C. (2020). Effects of somatostatin, curcumin, and quercetin on the fatty acid profile of breast cancer cell membranes. Can. J. Physiol. Pharmacol..

[10] Caro M., Sansone A., Amezaga J., Navarro V., Ferreri C., Tueros I. (2017). Wine lees modulate lipid metabolism and induce fatty acid remodelling in zebrafish. Food Funct..

[11] Hirata Y., Ferreri C., Yamada Y., Inoue A., Sansone A., Vetica F., Suzuki W., Takano S., Noguchi T., Matsuzawa A. (2023). Geometrical isomerization of arachidonic acid during lipid peroxidation interferes with ferroptosis. Free Radic. Biol. Med..

[12] Petran E.M., Periferakis A., Troumpata L., Periferakis A.T., Scheau A.E., Badarau I.A., Periferakis K., Caruntu A., Savulescu-Fiedler I., Sima R.M. (2024). Capsaicin: Emerging Pharmacological and Therapeutic Insights. Curr. Issues Mol. Biol..

[13] Scherer P.C., Zaccor N.W., Neumann N.M., Vasavda C., Barrow R., Ewald A.J., Rao F., Sumner C.J., Snyder S.H. (2017). TRPV1 is a physiological regulator of μ-opioid receptors. Proc. Natl. Acad. Sci. USA.

[14] Arora V., Campbell J.N., Chung M.K. (2021). Fight fire with fire: Neurobiology of capsaicin-induced analgesia for chronic pain. Pharmacol. Ther..

[15] Nisar A., Jagtap S., Vyavahare S., Deshpande M., Harsulkar A., Ranjekar P., Prakash O. (2023). Phytochemicals in the treatment of inflammation-associated diseases: The journey from preclinical trials to clinical practice. Front. Pharmacol..

[16] Zhang W., Zhang Y., Fan J.K., Feng Z.G., Song X.Q. (2024). Pharmacological activity of capsaicin: Mechanisms and controversies (Review). Mol. Med. Rep..

[17] Matsumoto T., Miyawaki C., Ue H., Yuasa T., Miyatsuji A., Moritani T. (2000). Effects of capsaicin-containing yellow curry sauce on sympathetic nervous system activity and diet-induced thermogenesis in lean and obese young women. J. Nutr. Sci. Vitamin..

[18] Cao Y.T., Xiang L.L., Qi F., Zhang Y.J., Chen Y., Zhou X.Q. (2022). Accuracy of controlled attenuation parameter (CAP) and liver stiffness measurement (LSM) for assessing steatosis and fibrosis in non-alcoholic fatty liver disease: A systematic review and meta-analysis. Eclinicalmedicine.

[19] Bort A., Sánchez B.G., Mateos-Gómez P.A., Díaz-Laviada I., Rodríguez-Henche N. (2019). Capsaicin Targets Lipogenesis in HepG2 Cells Through AMPK Activation, AKT Inhibition and PPARs Regulation. Int. J. Mol. Sci..

[20] Dashti N., Wolfbauer G. (1987). Secretion of lipids, apolipoproteins, and lipoproteins by human hepatoma cell line, HepG2: Effects of oleic acid and insulin. J. Lipid Res..

[21] Yao H.R., Liu J., Plumeri D., Cao Y.B., He T., Lin L., Li Y., Jiang Y.Y., Li J., Shang J. (2011). Lipotoxicity in HepG2 cells triggered by free fatty acids. Am. J. Transl. Res..

[22] Yang Y., Jiang Y., Wang Y., An W. (2010). Suppression of ABCA1 by unsaturated fatty acids leads to lipid accumulation in HepG2 cells. Biochimie.

[23] Gómez-Lechón M.J., Donato M.T., Martínez-Romero A., Jiménez N., Castell J.V., O’Connor J.E. (2007). A human hepatocellular in vitro model to investigate steatosis. Chem. Biol. Interact..

[24] Zheng J., Zheng S., Feng Q., Zhang Q., Xiao X. (2017). Dietary capsaicin and its anti-obesity potency: From mechanism to clinical implications. Biosci. Rep..

[25] Sharma N., Phan H.T.T., Yoda T., Shimokawa N., Vestergaard M.C., Takagi M. (2019). Effects of Capsaicin on Biomimetic Membranes. Biomimetics.

[26] Cui W., Chen S.L., Hu K.Q. (2010). Quantification and mechanisms of oleic acid-induced steatosis in HepG2 cells. Am. J. Transl. Res..

[27] Giampietri C., Petrungaro S., Cordella M., Tabolacci C., Tomaipitinca L., Facchiano A., Eramo A., Filippini A., Facchiano F., Ziparo E. (2017). Lipid Storage and Autophagy in Melanoma Cancer Cells. Int. J. Mol. Sci..

[28] Khalifa O., Mroue K.H., Mall R., Ullah E., Al-Akl N.S., Arredouani A. (2022). Investigation of the Effect of Exendin-4 on Oleic Acid-Induced Steatosis in HepG2 Cells Using Fourier Transform Infrared Spectroscopy. Biomedicines.

[29] Garcia C., Andersen C.J., Blesso C.N. (2023). The Role of Lipids in the Regulation of Immune Responses. Nutrients.

[30] She Y.B., Mangat R., Tsai S., Proctor S.D., Richard C. (2022). The Interplay of Obesity, Dyslipidemia and Immune Dysfunction: A Brief Overview on Pathophysiology, Animal Models, and Nutritional Modulation. Front. Nutr..

[31] Shimi G., Sohouli M.H., Ghorbani A., Shakery A., Zand H. (2024). The interplay between obesity, immunosenescence, and insulin resistance. Immun. Ageing.

[32] Gangabhagirathi R., Joshi R. (2015). Antioxidant activity of capsaicin on radiation-induced oxidation of murine hepatic mitochondrial membrane preparation. Res. Rep. Biochem..

[33] Müller F.A., Sturla S.J. (2019). Human in vitro models of nonalcoholic fatty liver disease. Curr. Opin. Toxicol..

[34] Fan H., Chen Y.Y., Bei W.J., Wang L.Y., Chen B.T., Guo J. (2013). In Vitro Screening for Antihepatic Steatosis Active Components within Coptidis Rhizoma Alkaloids Extract Using Liver Cell Extraction with HPLC Analysis and a Free Fatty Acid-Induced Hepatic Steatosis HepG2 Cell Assay. Evid. Based Complement. Altern. Med..

[35] Green C.J., Parry S.A., Gunn P.J., Ceresa C.D.L., Rosqvist F., Piché M.E., Hodson L. (2020). Studying non-alcoholic fatty liver disease: The ins and outs of in vivo, ex vivo and in vitro human models. Horm. Mol. Biol. Clin. Investig..

[36] Ramos M.J., Bandiera L., Menolascina F., Fallowfield J.A. (2022). In vitro models for non-alcoholic fatty liver disease: Emerging platforms and their applications. Iscience.

[37] Scanferlato R., Bortolotti M., Sansone A., Chatgilialoglu C., Polito L., De Spirito M., Maulucci G., Bolognesi A., Ferreri C. (2019). Hexadecenoic Fatty Acid Positional Isomers and De Novo PUFA Synthesis in Colon Cancer Cells. Int. J. Mol. Sci..

[38] Balint J.A., Kyriakides E.C., Beeler D.A. (1980). Fatty acid desaturation in lung: Inhibition by unsaturated fatty acids. J. Lipid Res..

[39] Inkpen C.A., Harris R.A., Quackenbush F.W. (1969). Differential responses to fasting and subsequent feeding by microsomal systems of rat liver: 6- and 9-desaturation of fatty acids. J. Lipid Res..

[40] Michaud M.R., Denlinger D.L. (2006). Oleic acid is elevated in cell membranes during rapid cold-hardening and pupal diapause in the flesh fly, *Sarcophaga crassipalpis*. J. Insect Physiol..

[41] Swain J., Mishra A.K. (2015). Location, Partitioning Behavior, and Interaction of Capsaicin with Lipid Bilayer Membrane: Study Using Its Intrinsic Fluorescence. J. Phys. Chem. B.

[42] Hanson S.M., Newstead S., Swartz K.J., Sansom M.S.P. (2015). Capsaicin Interaction with TRPV1 Channels in a Lipid Bilayer: Molecular Dynamics Simulation. Biophys. J..

[43] Cort A., Ozben T., Sansone A., Barata-Vallejo S., Chatgilialoglu C., Ferreri C. (2015). Bleomycin-induced trans lipid formation in cell membranes and in liposome models. Org. Biomol. Chem..

[44] Okada Y., Okajima H. (2001). Antioxidant effect of capsaicin on lipid peroxidation in homogeneous solution, micelle dispersions and liposomal membranes. Redox Rep..

[45] Vetica F., Sansone A., Meliota C., Batani G., Roberti M., Chatgilialoglu C., Ferreri C. (2020). Free-Radical-Mediated Formation of Trans-Cardiolipin Isomers, Analytical Approaches for Lipidomics and Consequences of the Structural Organization of Membranes. Biomolecules.

[46] Ferreri C., Ferocino A., Batani G., Chatgilialoglu C., Randi V., Riontino M.V., Vetica F., Sansone A. (2023). Plasmalogens: Free Radical Reactivity and Identification of Trans Isomers Relevant to Biological Membranes. Biomolecules.

[47] Shin S., Tae H., Park S., Cho N.J. (2023). Lipid Membrane Remodeling by the Micellar Aggregation of Long-Chain Unsaturated Fatty Acids for Sustainable Antimicrobial Strategies. Int. J. Mol. Sci..

[48] Wang Y.X., Jiang L., Shen Q.K., Shen J., Han Y.W., Zhang H.M. (2017). Investigation on the self-assembled behaviors of C18 unsaturated fatty acids in arginine aqueous solution. Rsc Adv..

[49] Zuo C., Zhang H., Liang S., Teng W., Bao C., Li D., Hu Y., Wang Q., Li Z., Li Y. (2021). The alleviation of lipid deposition in steatosis hepatocytes by capsaicin-loaded α-lactalbumin nanomicelles via promoted endocytosis and synergetic multiple signaling pathways. J. Funct. Foods.

[50] Lee Y.H., Kim H.J., You M., Kim H.A. (2022). Red Pepper Seeds Inhibit Hepatic Lipid Accumulation by Inducing Autophagy via AMPK Activation. Nutrients.

[51] Wu D., Duan R., Tang L., Zhou D.A., Zeng Z., Wu W., Hu J., Sun Q.M. (2022). In-vitro binding analysis and inhibitory effect of capsaicin on lipase. LWT.

[52] Munjuluri S., Wilkerson D.A., Sooch G., Chen X.J., White F.A., Obukhov A.G. (2022). Capsaicin and TRPV1 Channels in the Cardiovascular System: The Role of Inflammation. Cells.

[53] Zhang Q., Luo P., Xia F., Tang H., Chen J.Y., Zhang J.Z., Liu D.D., Zhu Y.P., Liu Y.Q., Gu L.W. (2022). Capsaicin ameliorates inflammation in a TRPV1-independent mechanism by inhibiting PKM2-LDHA-mediated Warburg effect in sepsis. Cell Chem. Biol..

[54] Al-Roub A., Al Madhoun A., Akhter N., Thomas R., Miranda L., Jacob T., Al-Ozairi E., Al-Mulla F., Sindhu S., Ahmad R. (2021). IL-1β and TNFα Cooperativity in Regulating IL-6 Expression in Adipocytes Depends on CREB Binding and H3K14 Acetylation. Cells.

[55] Saggini A., Anogeianaki A., Maccauro G., Teté S., Salini V., Caraffa A., Conti F., Fulcheri M., Galzio R., Shaik-Dasthagirisaheb Y.B. (2011). Cholesterol, cytokines and diseases. Int. J. Immunopathol. Pharmacol..

[56] Araujo P., Belghit I., Aarsæther N., Espe M., Lucena E., Holen E. (2019). The Effect of Omega-3 and Omega-6 Polyunsaturated Fatty Acids on the Production of Cyclooxygenase and Lipoxygenase Metabolites by Human Umbilical Vein Endothelial Cells. Nutrients.

[57] Mahmoud A.M., Mirza I., Metwally E., Morsy M.H., Scichilone G., Asada M.C., Mostafa A., Bianco F.M., Ali M.M., Masrur M.A. (2025). Lipidomic profiling of human adiposomes identifies specific lipid shifts linked to obesity and cardiometabolic risk. JCI Insight.

[58] Li R., Lan Y., Chen C., Cao Y., Huang Q., Ho C.T., Lu M. (2020). Anti-obesity effects of capsaicin and the underlying mechanisms: A review. Food Funct..

[59] Rizzo G., Baroni L., Lombardo M. (2023). Promising Sources of Plant-Derived Polyunsaturated Fatty Acids: A Narrative Review. Int. J. Environ. Res. Public Health.

[60] Sansone A., Melchiorre M., Chatgilialoglu C., Ferreri C. (2013). Hexadecenoic Fatty Acid Isomers: A Chemical Biology Approach for Human Plasma Biomarker Development. Chem. Res. Toxicol..

[61] Ferreri C., Sansone A., Ferreri R., Amézaga J., Tueros I. (2020). Fatty Acids and Membrane Lipidomics in Oncology: A Cross-Road of Nutritional, Signaling and Metabolic Pathways. Metabolites.

[62] Ferreri C., Sansone A., Buratta S., Urbanelli L., Costanzi E., Emiliani C., Chatgilialoglu C. (2020). The n-10 Fatty Acids Family in the Lipidome of Human Prostatic Adenocarcinoma Cell Membranes and Extracellular Vesicles. Cancers.

[63] Chiaradia E., Sansone A., Ferreri C., Tancini B., Latella R., Tognoloni A., Gambelunghe A., Dell’Omo M., Urbanelli L., Giovagnoli S. (2023). Phospholipid fatty acid remodeling and carbonylated protein increase in extracellular vesicles released by airway epithelial cells exposed to cigarette smoke extract. Eur. J. Cell Biol..

[64] Sansone A., Tolika E., Louka M., Sunda V., Deplano S., Melchiorre M., Anagnostopoulos D., Chatgilialoglu C., Formisano C., Di Micco R. (2016). Hexadecenoic Fatty Acid Isomers in Human Blood Lipids and Their Relevance for the Interpretation of Lipidomic Profiles. PLoS ONE.

[65] Ferreri C., Masi A., Sansone A., Giacometti G., Larocca A.V., Menounou G., Scanferlato R., Tortorella S., Rota D., Conti M. (2017). Fatty Acids in Membranes as Homeostatic, Metabolic and Nutritional Biomarkers: Recent Advancements in Analytics and Diagnostics. Diagnostics.

[66] Demirbolat G.M., Coskun G.P., Erdogan O., Cevik O. (2021). Long chain fatty acids can form aggregates and affect the membrane integrity. Colloids Surf. B Biointerfaces.

[67] Kay J.G., Fairn G.D. (2019). Distribution, dynamics and functional roles of phosphatidylserine within the cell. Cell Commun. Signal..

[68] Li Y.E., Norris D.M., Xiao F.N., Pandzic E., Whan R.M., Fok S., Zhou M., Du G.W., Liu Y., Du X.M. (2024). Phosphatidylserine regulates plasma membrane repair through tetraspanin-enriched macrodomains. J. Cell Biol..

[69] Huang S.P., Chen J.C., Wu C.C., Chen C.T., Tang N.Y., Ho Y.T., Lo C., Lin J.P., Chung J.G., Lin J.G. (2009). Capsaicin-induced Apoptosis in Human Hepatoma HepG2 Cells. Anticancer Res..

[70] Ibrahim M., Jang M., Park M., Gobianand K., You S., Yeon S.H., Park S., Kim M.J., Lee H.J. (2015). Capsaicin inhibits the adipogenic differentiation of bone marrow mesenchymal stem cells by regulating cell proliferation, apoptosis, oxidative and nitrosative stress. Food Funct..

[71] Mizogami M., Tsuchiya H. (2022). Membrane Interactivity of Capsaicin Antagonized by Capsazepine. Int. J. Mol. Sci..

[72] Nagy B., Fedonidis C., Photiou A., Wahba J., Paule C.C., Ma D., Buluwela L., Nagy I. (2009). Capsaicin-sensitive primary sensory neurons in the mouse express N-Acyl phosphatidylethanolamine phospholipase D. Neuroscience.

[73] Igarashi M., Iwasa K., Hayakawa T., Tsuduki T., Kimura I., Maruyama K., Yoshikawa K. (2023). Dietary oleic acid contributes to the regulation of food intake through the synthesis of intestinal oleoylethanolamide. Front. Endocrinol..

[74] Manchanda M., Leishman E., Sangani K., Alamri A., Bradshaw H.B. (2021). Activation of TRPV1 by Capsaicin or Heat Drives Changes in 2-Acyl Glycerols and N-Acyl Ethanolamines in a Time, Dose, and Temperature Dependent Manner. Front. Cell Develop. Biol..

[75] Kang J.H., Goto T., Han I.S., Kawada T., Kim Y.M., Yu R. (2010). Dietary capsaicin reduces obesity-induced insulin resistance and hepatic steatosis in obese mice fed a high-fat diet. Obesity.

[76] Tang J., Luo K., Li Y., Chen Q., Tang D., Wang D.M., Xiao J. (2015). Capsaicin attenuates LPS-induced inflammatory cytokine production by upregulation of LXRα. Int. Immunopharmacol..

[77] Poynter M.E., Mank M.M., Ather J.L. (2024). Obesity-associated inflammatory macrophage polarization is inhibited by capsaicin and phytolignans. Am. J. Physiol. Regul. Integr. Comp. Physiol..

[78] Reilly C.A., Taylor J.L., Lanza D.L., Carr B.A., Crouch D.J., Yost G.S. (2003). Capsaicinoids cause inflammation and epithelial cell death through activation of vanilloid receptors. Toxicol. Sci..

[79] Malagarie-Cazenave S., Olea-Herrero N., Vara D., Morell C., Díaz-Laviada I. (2011). The vanilloid capsaicin induces IL-6 secretion in prostate PC-3 cancer cells. Cytokine.

[80] Santa-María C., López-Enríquez S., Montserrat-de la Paz S., Geniz I., Reyes-Quiroz M.E., Moreno M., Palomares F., Sobrino F., Alba G. (2023). Update on Anti-Inflammatory Molecular Mechanisms Induced by Oleic Acid. Nutrients.

[81] Dushianthan A., Cusack R., Burgess V.A., Grocott M.P.W., Calder P.C. (2019). Immunonutrition for acute respiratory distress syndrome (ARDS) in adults. Cochrane Database Syst. Rev..

[82] Netea M.G., Dinarello C.A. (2011). More than Inflammation: Interleukin-1β Polymorphisms and the Lipid Metabolism. J. Clin. Endocrinol. Metab..

[83] Matsuki T., Horai R., Sudo K., Iwakura Y. (2003). IL-1 plays an important role in lipid metabolism by regulating insulin levels under physiological conditions. J. Exp. Med..

[84] Chatterjee P., Chiasson V.L., Bounds K.R., Mitchell B.M. (2014). Regulation of the ant-inflammatory cytokines interleukin-4 and interleukin-10 during pregnancy. Front. Immunol..

[85] Calder P.C. (2015). Functional Roles of Fatty Acids and Their Effects on Human Health. JPEN J. Parenter. Enter. Nutr..

[86] Honda K.L., Lamon-Fava S., Matthan N.R., Wu D.Y., Lichtenstein A.H. (2015). Docosahexaenoic acid differentially affects TNFα and IL-6 expression in LPS-stimulated RAW 264.7 murine macrophages. Prostaglandins Leukot. Essent. Fat. Acids.

[87] Castilla-Madrigal R., Gil-Iturbe E., de Calle M.L., Moreno-Aliaga M.J., Lostao M.P. (2020). DHA and its derived lipid mediators MaR1, RvD1 and RvD2 block TNF-α inhibition of intestinal sugar and glutamine uptake in Caco-2 cells. J. Nutr. Biochem..

[88] Hung H.C., Tsai S.F., Chou H.W., Tsai M.J., Hsu P.L., Kuo Y.M. (2023). Dietary fatty acids differentially affect secretion of pro-inflammatory cytokines in human THP-1 monocytes. Sci. Rep..

[89] So J., Wu D.Y., Lichtenstein A.H., Tai A.K., Matthan N.R., Maddipati K.R., Lamon-Fava S. (2021). EPA and DHA differentially modulate monocyte inflammatory response in subjects with chronic inflammation in part via plasma specialized pro-resolving lipid mediators: A randomized, double-blind, crossover study. Atherosclerosis.

[90] Calder P.C. (2013). Omega-3 polyunsaturated fatty acids and inflammatory processes: Nutrition or pharmacology?. Br. J. Clin. Pharmacol..

[91] Vetica F., Sansone A., Ferreri C., Chatgilialoglu C. (2022). A convenient route to mono-trans polyunsaturated free fatty acids. J. Chem. Res..

[92] Kohjima M., Enjoji M., Higuchi N., Kato M., Kotoh K., Nakashima M., Nakamuta M. (2009). The effects of unsaturated fatty acids on lipid metabolism in HepG2 cells. Vitr. Cell Dev. Biol. Anim..

[93] Folch J., Lees M., Sloane Stanley G.H. (1957). A simple method for the isolation and purification of total lipides from animal tissues. J. Biol. Chem..

[94] Bonaccio M., Di Castelnuovo A., Costanzo S., Ruggiero E., De Curtis A., Persichillo M., Tabolacci C., Facchiano F., Cerletti C., Donati M.B. (2019). Chili Pepper Consumption and Mortality in Italian Adults. J. Am. Coll. Cardiol..

