# Supplementary material for: Capsaicin and Its Combination with Oleic Acid Affect Membrane Fatty Acid Remodeling and Cytokine–Chemokine Secretion in HepG2 Cells
Source: Int J Mol Sci. 2025 Aug 25;26(17):8242. doi: 10.3390/ijms26178242 (PMC12428575; doi:10.3390/ijms26178242)
Supplement: Supplementary file 1 [file ijms-26-08242-s001.zip › ijms-3770496-supplementary.pdf]

Supplementary Information

Capsaicin and its combination with oleic acid affect membrane fatty a cid remodeling and cytokine-chemokine secretion in HepG2 cells

LIST of FATTY ACIDS

14:0 (myristic acid)
16:0 (palmitic acid)
trans 16:1
6 *cis* 16:1 (16:1 omega-10/ sapienic acid)
7 *cis* 16:1
9 *cis* 16:1 (palmitoleic acid)
18:0 (stearic acid)
9 *trans* 18:1 (elaidic acid)
8 *cis* 18:1 (18: 1 omega-10)
9 *cis* 18:1 (oleic acid)
11 *cis* 18:1 (*cis* vaccenic acid)
5 *cis*, 8 *cis* 18:2 (18:2 omega-10, sebaleic acid)
mono*trans* 18:2 omega-6 (9 *trans*, 12 *cis* 18:2)
18:2 omega-6 (9 *cis*, 12 *cis* 18:2, linoleic acid)
18:3 omega- 6 (6 *cis*, 9 cis, 12 cis/18:3) (gamma-linolenic acid)
18:3 omega-3 (9 *cis*, 12 *cis*, 15 *cis* 18:3) (alpha-linolenic acid)
11 *cis* 20:1 (gondoic acid)
20:2 omega-6, (11*cis*, 14 *cis* 20:2) (eicosadienoic acid)
20:3 omega-6 (8*cis*, 11*cis*, 14 *cis* 20:3) (DGLA, di-homo-gamma-linolenic acid)
20:4 omega-6 (5 *cis*, 8 *cis*,11 *cis*,14*cis* 20:4) (ARA, arachidonic acid)
mono*trans* ARA omega-6 (5 *trans*, 8 *cis*, 11 *cis*, 14 *cis* 20:4)
20:5 omega-3 (5 *cis,* 8 *cis*,11 *cis,*14 *cis*,17 *cis* 20:5) (EPA, eicosapentaenoic acid)
13 *cis* 22:1 (erucic acid)
22:5 omega-3 (7*cis*,10 *cis*,13 *cis*,16 *cis*,19 *cis* 22:5) (DPA, docosapentaenoic fatty acid)
22:6 omega-3, (4 *cis*, 7 *cis*,10 *cis*, 13 *cis*, 16 *cis*,19 *cis* 22:6) (DHA, docosahexaenoic fatty acid)
15 *cis* 24:1
total SFA (saturated fatty acids)
total MUFA (monounsaturated fatty acids)
total PUFA (polyunsaturated fatty acids)
total omega-6
total omega-3
omega-6/ omega-3
omega-10= sum of (6 *cis* 16:1+ 8 *cis* 16:1+ 8 *cis*, 5 *cis*, 18:2)
total *trans*

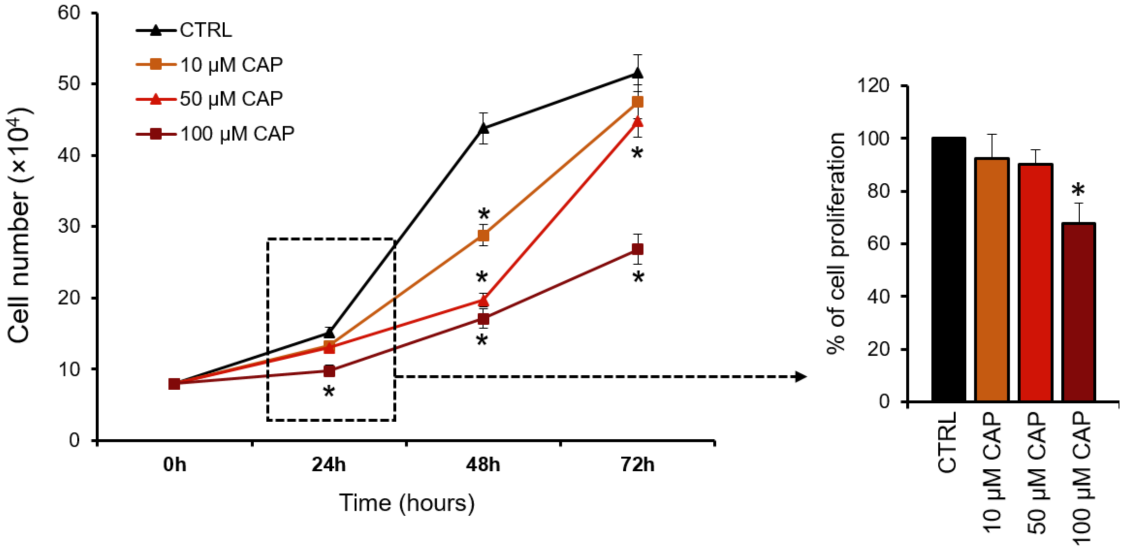

Figure S1. Capsaicin (CAP) affects HepG2 cell growth. Cell proliferation curve of HepG2 cells treated with 10, 50, and 100 µM CAP for 24, 48, and 72 h. The graphs represent the percentage of cell proliferation (CTRL=100%). Data represents the mean ± SD of three different experiments (statistical significance versus control: * $p \leq 0.05$).

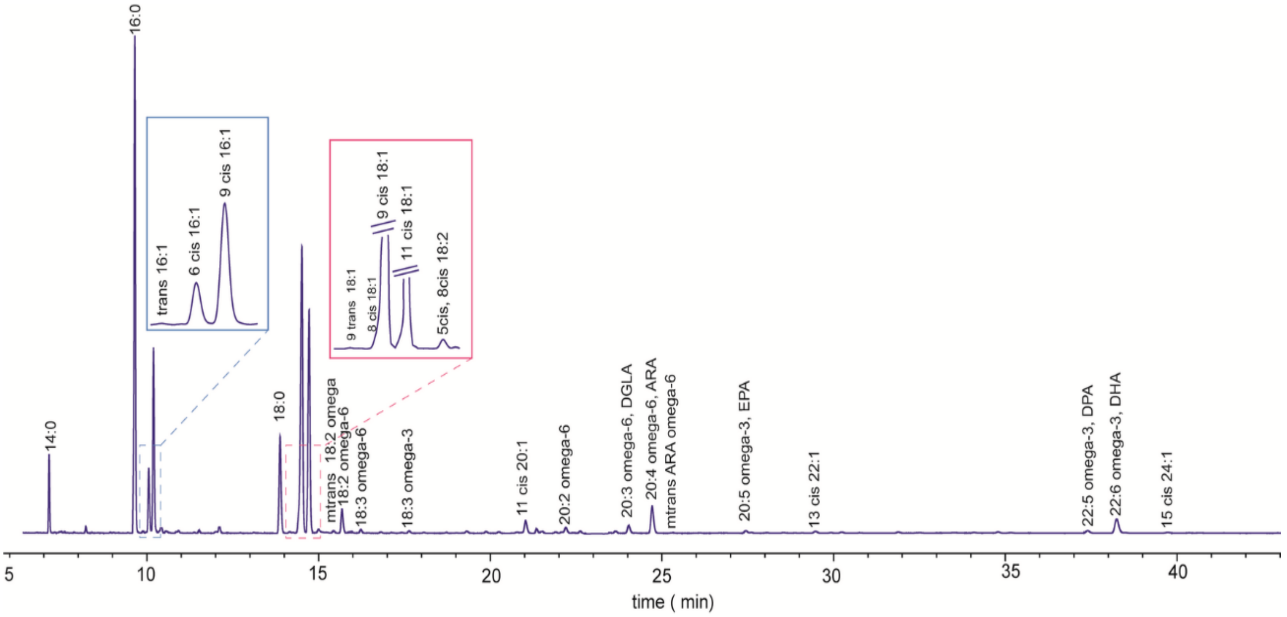

Figure S2. Representative GC chromatogram of the fatty acid methyl esters of HepG2 membrane phospholipids. The boxes show the expanded areas containing C16 MUFA (blue box) and 8*cis* C18:1 (18:1 omega-10), 5*cis*,8*cis* C18:2 (18:2 omega-10) (purple box)

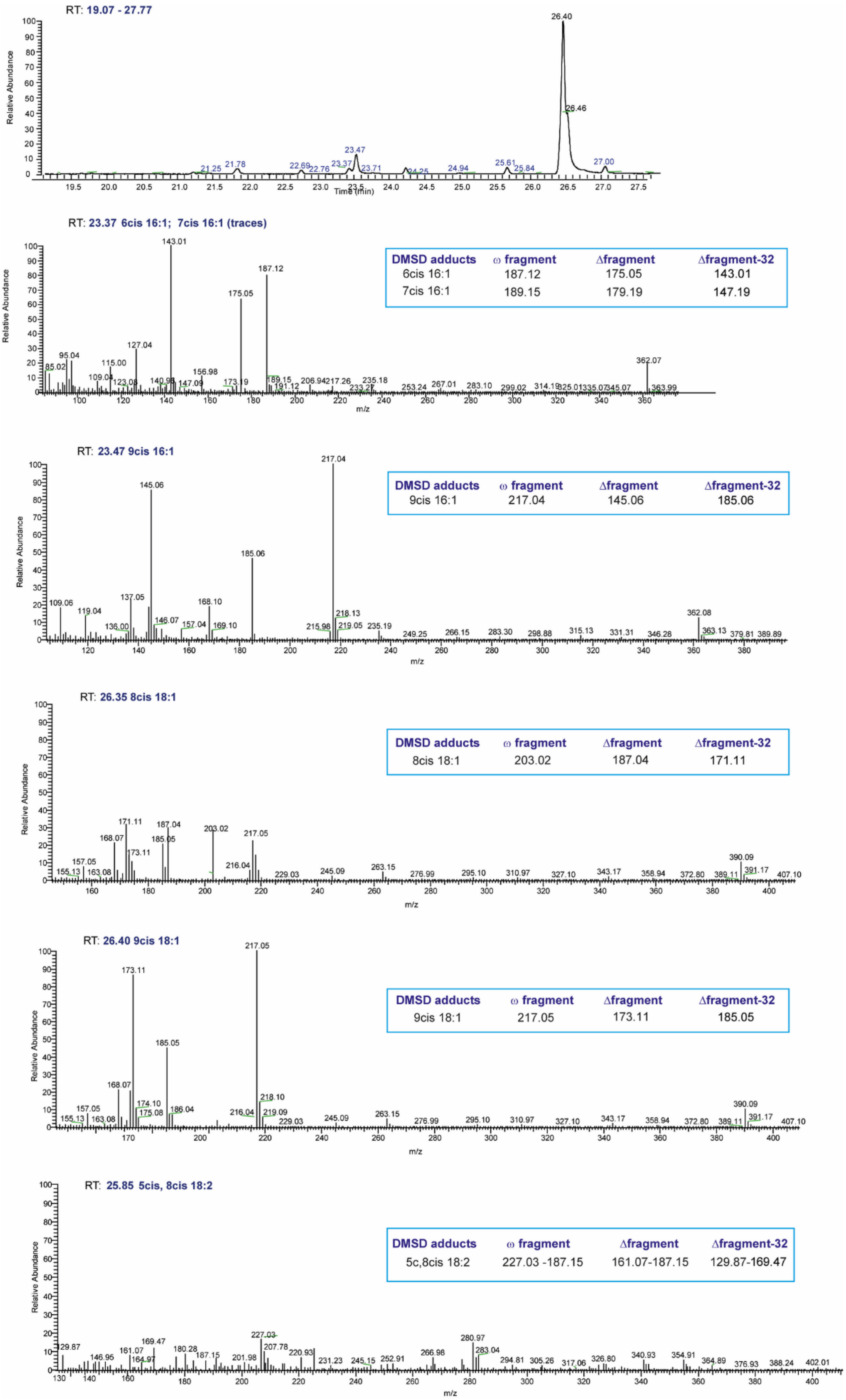

Figure S3. GC/MS analyses of FAME mixture obtained from the membrane phospholipids of the HepG2cell treated with OA and CAP after DMSD derivatization following the protocol described in Materials and Methods; GC/MS traces on the chromatographic region containing the FAME DMDS adducts of: 6*cis* C16:1 (16:1 omega-10), 9*cis* C16:1, 8*cis* C18:1 (18:1 omega-10), 9*cis* C18:1 and 5*cis*,8*cis* C18:2 (18:2 omega-10); the details of the diagnostic fragmentations of the DMDS adducts are showed in the blue boxes.

Table S1. FAME[1] (fatty acid methyl esters) obtained from the phospholipid membranes of HepG2 samples at 1.5h. They are expressed as relative percentages of the quantitative analyses of FAME calibrated with standard references in GC. The values are expressed as the mean±S.D. (standard deviation) of the experiments performed in triplicate; tr[2] (traces). Statistics was carried out by comparing each treatment with the control (CTRL) after 1.5h; significance: p values *: ≤ 0.05; ** ≤0.01; ***≤0.001; F≥4.7 for the significant p values.

Time 1.5h				
FAME[1] (% rel. quant.)	**CTRL**	**CAP**	**OA**	**OA+CAP**
14:0, myristic acid	2.19±0.53	1.93±0.56	2.69±0.28	3.67±0.31 **
16:0, palmitic acid	28.08±1.86	25.31±1.76	24.62±1.41	23.05±0.50 **
trans 16:1	0.12±0.01	0.12±0.01	0.12±0.01	0.12±0.01
6 *cis* 16:1, 16:1 omega-10, sapienic acid	2.78±0.48	2.93±0.28	3.19±0.43	3.27±0.20
7 *cis* 16:1	tr[2]	tr[2]	tr[2]	tr[2]
9 *cis* 16:1 , palmitoleic acid	6.88±0.96	7.71±1.08	8.13±1.03	8.66±0.37
18:0, stearic acid	8.76±0.89	7.30±1.89	6.14±0.39*	5.82±0.08 *
9 *trans* 18:1, elaidic acid	0.09±0.07	0.07±0.06	0.13±0.08	0.14±0.00
8 *cis* 18:1 omega-10	0.94±0.11	1.10±0.23	1.03±0.07	1.01±0.11
9 *cis* 18:1, oleic acid	22.46±0.18	23.32±0.99	26.57±0.38***	26.06±1.14**
11 *cis* 18:1, cis vaccenic acid	13.35±1.11	14.76±0.24	12.36±0.45	12.44±0.30
5 *cis*, 8 *cis* 18:2, 18:2 omega-10, sebaleic acid	0.56±0.07	0.56±0.05	0.57±0.02	0.61±0.08
mono*trans* 18:2 omega-6	0.06±0.03	0.06±0.01	0.07±0.02	0.10±0.09
18:2 omega-6, linoleic acid	1.71±0.41	1.45±0.16	1.47±0.21	1.39±0.06
18:3 omega-6, gamma linolenic acid	0.46±0.05	0.45±0.01	0.55±0.13	0.77±0.69
18:3 omega-3, alfa linolenic acid	0.37±0.08	0.33±0.07	0.59±0.19	0.51±0.03
11 *cis* 20:1, gondoic acid	1.29±0.12	1.38±0.07	1.36±0.11	1.42±0.14
20:2 omega-6, eicosadienoic acid	0.57±0.07	0.51±0.08	0.61±0.06	0.59±0.05
20:3 omega-6, DGLA	1.76±0.11	1.70±0.70	1.55±0.03	1.72±0.27
20:4 omega-6, ARA	2.62±0.10	3.24±0.56	2.82±0.34	2.83±0.25
mono*trans* ARA	0.04±0.02	0.03±0.01	0.05±0.00	0.04±0.00
20:5 omega-3, EPA	0.50±0.08	0.63±0.04	0.60±0.04	0.64±0.05
13 *cis* 22:1	0.53±0.14	0.50±0.02	0.51±0.08	0.52±0.01
22:5 omega-3, DPA	0.61±0.12	0.72±0.10	0.79±0.06	0.69±0.07
22:6 omega-3, DHA	2.06±0.39	2.17±0.75	2.34±0.28	2.54±0.41
15 *cis* 24:1	0.39±0.08	0.35±0.08	0.46±0.08	0.41±0.04
total SFA	39.03±2.34	34.55±3.28	33.45±1.74*	32.54±0.34*
total MUFA	48.62±2.55	52.06±0.91	53.61±1.54*	53.78±0.73*
total PUFA	11.22±0.32	11.78±2.16	11.89±0.53	12.29±0.97
total omega-6	7.12±0.37	7.36±1.23	6.99±0.26	7.30±0.61
total omega-3	3.54±0.67	3.86±0.91	4.33±0.28	4.38±0.48
omega-6/ omega-3	2.08±0.55	1.93±0.13	1.62±0.06	1.67±0.18
omega-10	4.28±0.49	4.59±0.38	4.79±0.35	4.88±0.33
total *trans*	0.30±0.11	0.29±0.05	0.37±0.07	0.39±0.10

Table S2. FAME[1] (fatty acid methyl esters) obtained from the phospholipid membranes of HepG2 samples at 3h. They are expressed as relative percentages of the quantitative analyses of FAME calibrated with standard references in GC. The values are expressed as the mean±S.D. (standard deviation) of the experiments performed in triplicates; tr[2] (traces). Statistics were carried out by comparing each treatment with the control (CTRL) after 3 h; significance: p values *: ≤ 0.05; ** ≤0.01; ***≤ 0.001; F≥4.46 for the significant p values.

	**Time 3 h**			
FAME[1] (%rel .quant.)	**CTRL**	**CAP**	**OA**	**OA+CAP**
14:0, myristic acid	2.56±0.12	2.39±0.62	2.10±0.19	1.97±0.02
16:0, palmitic acid	24.18±0.72	23.84±1.05	23.18±0.48	21.93±0.66*
trans 16:1	0.05±0.03	0.08±0.00	0.11±0.03	0.11±0.03
6 *cis* 16:1, 16:1 omega-10, sapienic acid	3.19±0.05	2.89±0.18 *	2.93±0.08	2.46±0.10***
7 *cis* 16:1	tr[2]	tr[2]	tr[2]	tr[2]
9 *cis* 16:1 , palmitoleic acid	8.00±0.28	7.63±0.32	6.69±0.23***	5.89±0.10***
18:0, stearic acid	6.84±0.09	7.49±0.57	6.36±0.41	5.95±0.35
9 *trans* 18:1, elaidic acid	0.23±0.15	0.17±0.02	0.11±0.06	0.13±0.03
8 *cis* 18:1 omega-10	1.03±0.09	1.05±0.17	1.17±0.22	1.06±0.15
9 *cis* 18:1, oleic acid	22.29±0.60	23.56±0.41	27.97±0.49***	31.00±0.91***
11 *cis* 18:1, cis vaccenic acid	15.47±0.17	14.85±0.61	13.70±0.25**	13.31±0.50***
5 *cis*, 8 *cis* 18:2, 18:2 omega-10, sebaleic acid	0.71±0.04	0.62±0.04	0.67±0.07	0.65±0.02
mono*trans* 18:2 omega-6	0.23±0.28	0.05±0.01	0.05±0.00	0.05±0.00
18:2 omega-6, linoleic acid	1.50±0.02	1.37±0.06*	1.39±0.04	1.23±0.07***
18:3 omega-6, gamma linolenic acid	0.51±0.09	0.49±0.06	0.48±0.06	0.51±0.06
18:3 omega-3, alfa linolenic acid	0.51±0.05	0.49±0.05	0.54±0.04	0.60±0.09
11 *cis* 20:1, gondoic acid	1.45±0.06	1.40±0.16	1.56±0.16	1.77±0.08*
20:2 omega-6, eicosadienoic acid	0.67±0.03	0.70±0.05	0.74±0.02	0.69±0.03
20:3 omega-6, DGLA	1.92±0.22	2.00±0.19	1.94±0.39	1.90±0.16
20:4 omega-6, ARA	3.00±0.36	2.76±0.12	2.82±0.18	2.68±0.18
mono*trans* ARA	0.19±0.27	0.05±0.00	0.04±0.00	0.04±0.00
20:5 omega-3, EPA	0.64±0.06	0.61±0.06	0.60±0.06	0.68±0.04
13 *cis* 22:1	0.53±0.05	0.63±0.07	0.63±0.11	0.64±0.01
22:5 omega-3, DPA	0.72±0.10	0.73±0.03	0.75±0.04	0.76±0.05
22:6 omega-3, DHA	2.36±0.21	2.61±0.25	2.17±0.10	2.23±0.09
15 *cis* 24:1	0.46±0.06	0.46±0.10	0.46±0.03	0.43±0.01
total SFA	33.58±0.94	33.72±1.73	31.65±0.56	29.85±0.32**
total MUFA	52.43±0.66	52.47±1.55	55.11±0.45*	56.56±0.30**
total PUFA	12.53±0.81	12.40±0.53	12.10±0.74	11.93±0.24
total omega-6	7.61±0.63	7.33±0.28	7.37±0.61	7.01±0.09
total omega-3	4.22±0.20	4.44±0.27	4.05±0.20	4.27±0.20
omega-6/ omega-3	1.80±0.11	1.65±0.05	1.82±0.15	1.65±0.07
omega-10	4.92±0.09	4.57±0.37	4.78±0.29	4.17±0.24*
total *trans*	0.93±0.84	0.52±0.03	0.42±0.14	0.47±0.03

Table S3. FAME[1] (fatty acid methyl esters) obtained from the phospholipid membranes of HepG2 samples at 24h.They are expressed as relative percentages of the quantitative analyses of FAME recognized and calibrated with standard references in GC. The values are expressed as the mean±S.D. (standard deviation) of the experiments performed in triplicate. tr[2] (traces). Statistics were carried out by comparing each treatment with the control (CTRL) after 24h; significance p values *:≤ 0.05; **≤ 0.01; ***≤0.001; **** ≤0.0001; F≥6.80 for the significant p values.

Time 24 h				
FAME[1] (%rel quant)	CTRL	CAP	AO	AO+CAP
14:0, myristic acid	2.97±0.14	2.60±0.18	2.57±0.09	1.84±0.28***
16:0, palmitic acid	26.02±0.73	25.60±1.59	24.02±0.37	30.30±1.97*
trans 16:1	0.09±0.01	0.09±0.01	0.08±0.01	0.06±0.01***
6 *cis* 16:1, 16:1 omega-10 /sapienic acid	3.67±0.27	3.26±0.22	2.51±0.16**	2.01±0.31**
7 *cis* 16:1	tr[2]	tr[2]	tr[2]	tr[2]
9 *cis* 16:1 , palmitoleic acid	7.17±0.62	7.77±0.22	4.75±0.30***	3.49±0.14***
18:0, stearic acid	6.55±0.40	6.64±0.25	6.54±0.33	4.86±0.43**
9 *trans* 18:1, elaidic acid	0.24±0.18	0.09±0.05	0.11±0.03	0.18±0.09
8 *cis* 18:1 omega-10	1.03±0.10	1.34±0.16	1.17±0.22	1.15±0.12
9 *cis* 18:1, oleic acid	22.14±0.50	22.03±1.66	33.44±1.21***	36.14±0.79***
11 *cis* 18:1, cis vaccenic acid	13.48±0.64	14.16±1.08	9.35±0.57***	7.40±0.83****
5 *cis*, 8 *cis* 18:2, 18:2 omega-10, sebaleic acid	0.69±0.03	0.67±0.04	0.62±0.11	0.45±0.08**
mono*trans* 18:2 omega-6	0.19±0.24	0.06±0.01	0.05±0.00	0.05±0.01
18:2 omega-6, linoleic acid	1.34±0.07	1.52±0.09	1.30±0.15	1.07±0.06*
18:3 omega-6, gamma linolenic acid	0.46±0.03	0.55±0.07	0.50±0.10	0.37±0.0
18:3 omega-3, alfa linolenic acid	0.49±0.09	0.57±0.08	0.50±0.09	0.36±0.08
11 *cis* 20:1, gondoic acid	1.36±0.08	1.31±0.12	1.96±0.19**	1.86±0.17**
20:2 omega-6, eicosadienoic acid	0.71±0.07	0.74±0.05	0.74±0.09	0.57±0.11
20:3 omega-6, DGLA	1.99±0.21	2.00±0.17	1.68±0.12	1.38±0.15**
20:4 omega-6, ARA	3.10±0.29	2.95±0.29	2.55±0.46	1.94±0.33**
mono*trans* ARA	0.16±0.19	0.05±0.01	0.04±0.01	0.03±0.01
20:5 omega-3, EPA	0.65±0.10	0.63±0.14	0.60±0.13	0.43±0.06
13 *cis* 22:1	0.57±0.07	0.58±0.04	0.62±0.05	0.40±0.12
22:5 omega-3, DPA	0.75±0.07	0.75±0.11	0.68±0.08	0.48±0.05**
22:6 omega-3, DHA	2.80±0.24	2.74±0.17	2.02±0.21**	1.70±0.16***
15 *cis* 24:1	0.49±0.06	0.49±0.06	0.52±0.08	0.36±0.06
total SFA	35.53±0.21	34.84±1.40	33.14±0.51*	36.99±1.29
total MUFA	49.90±0.91	50.95±0.15	54.33±0.13***	52.81±0.72**
total PUFA	12.98±0.85	13.12±1.01	11.21±1.42	8.76±0.85**
total omega-6	7.60±0.48	7.76±0.58	6.79±0.85	5.34±0.48**
total omega-3	4.69±0.36	4.69±0.42	3.80±0.49	2.98±0.33**
omega-6/ omega-3	1.62±0.03	1.66±0.06	1.79±0.10*	1.79±0.04*
omega-10	5.39±0.22	5.27±0.36	4.30±0.41*	3.61±0.44***
total *trans*	0.92±0.79	0.37±0.10	0.39±0.07	0.51±0.17

Table S4. Incubation of linoleic acid methyl ester in solution. The PUFA loss is calculated by considering the quantity of linoleic methyl ester at starting time without incubation

Incubation of linoleic acid methyl ester in isopropanol solution 37°C, 6 h	PUFA loss%* (= linoleic acid methyl ester loss)
+ 10 μM CAP	0%
+ 100 μM OA	3%
mixture 10 μM CAP + 100 μM OA	0%

Table S5. FAME[1] (fatty acid methyl esters) obtained from the triglycerides after 3 hours of treatment with OA (100 µM) and OA and CAP (100µM+10µM). They are expressed as the relative percentages of the quantitative analyses of FAME calibrated with standard references in GC. The values are expressed as the mean±S.D. (standard deviation) of the experiments performed in triplicate; tr[2] (traces); nd[3] (not detected); . Statistics were carried out by comparing the OA and CAP treatment with OA used as a control; p values *: ≤0.05; *: ≤0.05 (t value ≥2.91-3.16); **: ≤0.01(t value ≥4.74-5.08); ***≤0.001(t value ≥11.69).

FAME[1] (%rel quant)	OA<br>TG 3h	OA+CAP<br>TG 3h
14:0, myristic acid	2.81±0.50	3.02±0.27
16:0, palmitic acid	32.22±1.03	31.33±0.64
trans 16:1	tr[2]	tr[2]
6 *cis* 16:1, 16:1 omega-10, sapienic acid	2.18±0.12	2.67±0.23*
7 *cis* 16:1	tr[2]	tr[2]
9 *cis* 16:1 , palmitoleic acid	6.41±0.07	6.33±0.20
18:0, stearic acid	5.13±0.08	6.16±0.13***
9 *trans* 18:1, elaidic acid	0.07±0.04	0.08±0.04
8 *cis* 18:1 omega-10	1.10±0.03	1.21±0.18
9 *cis* 18:1, oleic acid	26.17±0.74	24.51±0.53*
11 *cis* 18:1, cis vaccenic acid	18.17±0.48	16.54±0.28**
5 *cis*, 8 *cis* 18:2, 18:2 omega-10, sebaleic acid	0.23±0.16	0.43±0.02
mono*trans* 18:2 omega-6	nd[3]	nd[3]
18:2 omega-6, linoleic acid	1.19±0.39	1.07±0.22
18:3 omega-6, gamma linolenic acid	0.02±0.02	0.08±0.04
18:3 omega-3, alfa linolenic acid	0.21±0.08	0..098±0.05
11 *cis* 20:1, gondoic acid	1.61±0.20	1.55±0.16
20:2 omega-6, eicosadienoic acid	0.12±0.03	tr[2]
20:3 omega-6, DGLA	0.18±0.17	0.72±0.10**
20:4 omega-6, ARA	1.31±0.15	1.22±0.47
mono*trans* ARA	nd[3]	nd[3]
20:5 omega-3, EPA	0.14±0.03	0.24±0.11
13 *cis* 22:1	0.33±0.11	0.41±0.17
22:5 omega-3, DPA	0.21±0.16	0.23±0.27
22:6 omega-3, DHA	0.71±0.27	0.53±0.32
15 *cis* 24:1	0.00±0.00	0.08±0.13
total SFA	40.17±0.61	40.51±0.68
total MUFA	55.97±0.98	53.44±0.62*
total PUFA	4.63±0.10	4.99±1.33
total omega-6	2.83±0.49	3.20±0.72
total omega-3	1.28±0.51	1.07±059
omega-6/ omega-3	2.70±1.43	3.50±1.81
omega-10	3.52±0.26	4.28±0.37*
total *trans*	0.09±0.06	0.09±0.13

Table S6. FAME[1] (fatty acid methyl esters) obtained from the triglycerides after 24 hours of treatment with OA (100μM) and OA and CAP (100μM+10μM). They are expressed as the relative percentages of the quantitative analyses of FAME calibrated with standard references in GC. The values are expressed as the mean±S.D. (standard deviation) of the experiments performed in triplicate; tr[2] (traces); nd[3] (not detected). Statistics were carried out comparing the OA and CAP treatment with OA used as control; p values *: ≤0.5 (t value ≥3.0-3.78); **: ≤0.01 (t value ≥5.2-7.13); ***≤0.001(t value =11.9).

FAME[1] (%rel quant)	OA<br>TG 24 h	OA+CAP<br>TG 24 h
14:0, myristic acid	2.74±0.02	3.24±0.07***
16:0, palmitic acid	29.93±0.29	29.49±0.57
trans 16:1	nd	nd
6 *cis* 16:1, 16:1 omega-10, sapienic acid	1.97±0.02	2.23±0.20
7 *cis* 16:1	tr[2]	tr[2]
9 *cis* 16:1 , palmitoleic acid	3.94±0.07	3.59±0.21
18:0, stearic acid	4.78±0.61	7.51±0.25**
9 *trans* 18:1, elaidic acid	0.05±0.05	0.04±0.01
8 *cis* 18:1 omega-10	0.62±0.27	0.76±0.10
9 *cis* 18:1, oleic acid	39.61±0.57	37.38±0.53**
11 *cis* 18:1, cis vaccenic acid	10.62±0.37	9.10±0.34**
5 *cis*, 8 *cis* 18:2, 18:2 omega-10, sebaleic acid	0.13±0.06	0.26±0.16
mono*trans* 18:2 omega-6	nd	nd
18:2 omega-6, linoleic acid	0.65±0.06	0.79±0.20
18:3 omega-6, gamma linolenic acid	nd	nd
18:3 omega-3, alfa linolenic acid	nd	nd
11 *cis* 20:1, gondoic acid	2.03±0.01	2.13±0.10
20:2 omega-6, eicosadienoic acid	0.22±0.10	0.03±0.02
20:3 omega-6, DGLA	0.36±0.01	0.37±0.11
20:4 omega-6, ARA	0.86±0.19	0.68±0.21
mono*trans* ARA	0.02±0.00	0.02±0.00
20:5 omega-3, EPA	0.23±0.15	0.28±0.01
13 *cis* 22:1	0.19±0.07	0.42±0.14
22:5 omega-3, DPA	0.18±0.01	0.34±0.17
22:6 omega-3, DHA	0.85±0.21	0.46±0.08*
15 *cis* 24:1	0.11±0.02	0.01±0.01
total SFA	37.44±0.91	40.24±0.87*
total MUFA	59.09±0.64	55.47±0.52**
total PUFA	3.67±0.39	3.29±0.51
total omega-6	2.08±0.32	1.87±0.25
total omega-3	1.27±0.19	0.88±0.29
omega-6/ omega-3	1.67±0.42	2.36±1.03
omega-10	2.72±0.34	3.25±0.34
total *trans*	0.07±0.04	0.04±0.01
